# Enhancing the Implant Osteointegration via Supramolecular Co‐Assembly Coating with Early Immunomodulation and Cell Colonization

**DOI:** 10.1002/advs.202410595

**Published:** 2025-01-13

**Authors:** Chenglong Wang, Zeyu Shou, Chengwei Xu, Kaiyuan Huo, Wenjie Liu, Hao Liu, Xingjie Zan, Qing Wang, Lianxin Li

**Affiliations:** ^1^ Department of Orthopaedics Surgery Shandong Provincial Hospital Affiliated to Shandong First Medical University Jinan Shandong 250021 China; ^2^ Department of Orthopedics Zhuji People's Hospital of Zhejiang Province Zhuji Affiliated Hospital of Wenzhou Medical University Shaoxing Zhejiang 311800 China; ^3^ Department of Orthopedics The First Affiliated Hospital of Wenzhou Medical University Wenzhou Zhejiang 325000 China; ^4^ Wenzhou Institute University of Chinese Academy of Sciences Wenzhou Key Laboratory of Perioperative Medicine Wenzhou Zhejiang 325001 China; ^5^ School of Materials Science and Engineering Zhengzhou University Zhengzhou 450001 China; ^6^ Yongkang First People's Hospital of Wenzhou Medical University Jinhua 321300 China

**Keywords:** colonization, metal‐phenolic network, osteoimmunology, RGD, strontium

## Abstract

Osteointegration, the effective coupling between an implant and bone tissue, is a highly intricate biological process. The initial stages of bone‐related immunomodulation and cellular colonization play crucial roles, but have received limited attention. Herein, a novel supramolecular co‐assembled coating of strontium (Sr)‐doped metal polyphenol networks (MPN) modified with c(RGDfc) is developed and well‐characterized, for eliciting an early immunomodulation and cellular colonization. The results showed that the (Sr‐MPN)@RGD coating significantly regulated the polarization of macrophages to the M2 phenotype by controllable release of Sr, and promote the initial adhesion of bone marrow mesenchymal stem cells (BMSCs) by RGD presented on MPN. Notably, the (Sr‐MPN)@RGD attenuated osteoclast differentiation and oxidative stress as well as enhanced osteoblast differentiation and angiogenesis due to macrophage polarization toward M2 phenotype, which in turn has a profound effect on neighboring cells through paracrine signaling. In vivo results showed that the (Sr‐MPN)@RGD coating manifested superior osseointegration and bone maturation to the bare Ti‐rod or Ti‐rod coated with MPN and Sr‐MPN. This work contributed to the design of multifunctional implant coatings that address the complex biological process of osteointegration from the perspective of orchestrating stem cell recruitment with immunomodulatory strategies.

## Graphical abstract

The (Sr‐MPN)@RGD coating, combining Sr‐doped metal polyphenol networks and cRGD modification, enhances implant osteointegration by reducing osteoclast differentiation, oxidative stress, and promoting osteoblast differentiation, angiogenesis, and M2 macrophage polarization. In vivo, it outperformed bare Ti‐rod and Ti‐rod coated with MPN or Sr‐MPN in osseointegration and bone maturation. This approach integrates stem cell recruitment and immunomodulation for improved osteointegration.

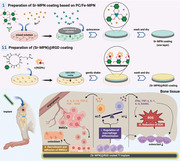

## 1 Introduction

The repair of injured bone is a complex and orderly biological process encompassing various stages such as cell recruitment, inflammatory response, fibrovascular formation, bone formation, and remodeling. These biological processes occur sequentially and intertwine, collectively contributing to the restoration of bone tissue.^[^ ^1^, ^2^ ^]^ Notably, the biological processes in the early stages are crucial in determining the success of bone injury repair.^[^ ^3^ ^]^ Shortly after the implantation of bone implants, a blood clot rapidly forms around the damaged bone tissue, while the inflammatory response is promptly activated. Inflammatory cells, such as macrophages and neutrophils, migrate to the implant site, eliminating necrotic tissue and foreign bodies while secreting various cytokines.^[^ ^4^, ^5^ ^]^ Concurrently, bone marrow mesenchymal stem cells (BMSCs) are attracted by chemokines and adhere to the implant surface or extracellular matrix through adhesion molecules, eventually colonizing the implant surface or surrounding tissues.^[^ ^6^ ^]^ These stem cells possess multilineage differentiation potential, capable of differentiating into osteoblasts that synthesize and secrete bone matrix. Further mineralization deposits inorganic salts such as calcium and phosphates into the bone matrix, ultimately leading to the formation of rigid bone tissue.^[^ ^7^ ^]^ Therefore, early inflammatory regulation and cell recruitment processes significantly impact bone formation during the healing of injured bone.

However, implants, typically represented by titanium (Ti) and titanium alloys, act as foreign bodies that recruit immune cells and trigger inflammation (known as peri‐implantitis).^[^ ^8^, ^9^ ^]^ In the inflammatory microenvironment, excessive accumulation of inflammatory factors and the presence of reactive oxygen species may impair the secretion of growth factors. Additionally, the bioinertness of Ti and titanium alloys makes the adhesion, colonization, and growth of BMSCs challenging. These factors lead to reduced osteogenic differentiation of bone tissue, decreased angiogenesis, and excessive activation of osteoclasts, which ultimately contribute to poor bone healing and implant failure.^[^ ^10^ ^]^ Consequently, the immunomodulatory and cell colonization capabilities of biomaterials hold significant importance in bone repair but have received limited attention.^[^ ^11^ ^]^

Macrophages, as a crucial component of the bone immunological microenvironment, play a pivotal role in early bone injury repair and host defense reactions to implants.^[^ ^12^ ^]^ Macrophages can generally be classified into two subtypes: M1 and M2. Overactivation of the M1 subtype often promotes inflammation and leads to fibrotic encapsulation, whereas activation of the M2 subtype can facilitate tissue regeneration by secreting anti‐inflammatory and pro‐healing cytokines.^[^ ^13^ ^]^ The ratio of M1 to M2 subtypes significantly modulates bone regeneration during the process. This ratio can be modulated by external environmental stimuli, such as proteins (IL‐4 and BMP‐2),^[^ ^14^ ^]^ polyphenols (TA, PC),^[^ ^15^ ^]^ and metal ions (Zn^2+^, Sr^2+^, and Cu^2+^).^[^ ^16^ ^]^ Among them, metal ions, especially Sr^2+^, have been used as part of the raw materials for the design of a variety of biomaterials, and have been proved to inhibit the M1 differentiation of macrophages and promote the differentiation of M2 subtypes through a variety of potential mechanisms, thereby regulating the bone immune microenvironment.^[^ ^17^ ^]^ Moreover, due to its unique osteogenic ability, Sr has been widely used in the design of bone biomaterials.^[^ ^18^ ^]^

Bone implant materials that enhance stem cell recruitment and colonization are crucial for bone repair. Successfully recruited and colonized BMSCs can differentiate into osteoblasts, which synthesize and secrete bone matrix that mineralizes to form rigid bone tissue. Most research focuses on incorporating bioactive factors (e.g., growth factors^[^ ^19^ ^]^) and extracellular matrix proteins^[^ ^20^ ^]^ into material surfaces or structures. For instance, Bai et al. developed collagen and proanthocyanidin coatings that significantly improved BMSC adhesion and osteogenic differentiation.^[^ ^21^ ^]^ Previously, our research group has developed a strategy to efficiently immobilize the cell‐adhesive short peptide RGD onto MPN coatings through a click chemistry reaction between thiol groups and double bonds, significantly enhancing cell adhesion, migration, and proliferation activities.^[^ ^22^, ^23^ ^]^ These materials mimic the natural bone tissue microenvironment, providing favorable conditions for BMSC recruitment and colonization.^[^ ^24^, ^25^ ^]^ On a deeper level, the initial cell recruitment and colonization processes significantly influence subsequent signal transduction. BMSC colonization triggers signaling cascades that activate growth factor and cytokine secretion, regulate extracellular matrix protein expression, and modulate cell‐cell interactions. These mechanisms promote osteogenic gene expression, regulate bone calcification, and enhance angiogenesis. Thus, efficient cell recruitment and colonization together with beneficial immune regulation create a local microenvironment conducive to bone formation.

Polyphenol and metal are stably chelated through supramolecular interaction to form metal polyphenol network (MPN).^[^ ^26^ ^]^ This unique structure allows MPN to synergistically integrate the biological functions of both polyphenols and metal ions, offering a versatile platform for bioactive coatings. It exhibits significant potential in maintaining the biological characteristics of its constituent components^[^ ^27^ ^]^ and offers numerous advantages, including simplicity in preparation, adhesion to substrate materials regardless of the shape of the implant, and excellent controllability of deposition thickness at the nanoscale.^[^ ^26^ ^]^ In our previous study, we deeply investigated the MPN coating based on proanthocyanidin (PC) and Fe (III), and we found that this coating has strong antioxidant capacity, which can effectively scavenge reactive oxygen species (ROS) and mitigate the local microenvironment.^[^ ^28^, ^29^ ^]^ This ROS‐scavenging ability, coupled with the inherent anti‐inflammatory properties of many metals, such as Sr, can create a favorable immunological milieu around the implant, potentially enhancing bone integration and repair processes. Unfortunately, we have found that Sr‐doped MPN coatings lack the ability to promote stem cell recruitment due to the absence of cell adhesion sites.^[^ ^22^ ^]^ Therefore, a strategy that synergistically integrates bone immune regulation and cell recruitment based on the critical early repair processes in injured bone remains highly worth exploring.

**Scheme 1:**
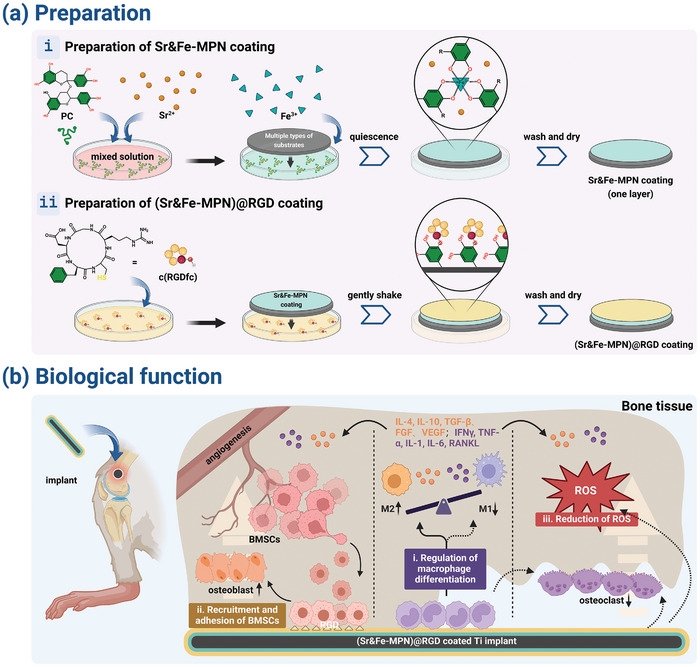
a) Schematic representation of the workflow of (i) Sr‐MPN coating and (ii) (Sr‐MPN)@RGD coating. b) The biological function of (Sr‐MPN)@RGD coating. (i) Primarily, it modulates macrophage polarization, favoring the M1 phenotype over the M2 phenotype. (ii) This coating demonstrates a capacity to recruit BMSCs to its surface, significantly enhancing cellular adhesion. (iii) The coating showcases potent antioxidant properties, effectively scavenging reactive oxygen species (ROS). These combined functionalities culminate in the promotion of surrounding bone and vascular tissue formation while simultaneously inhibiting bone resorption.

In this work, we doped Sr into the MPN coating formed by PC and Fe (III) (noted as (Sr‐MPN), then fixed RGD peptides onto MPN (noted as (Sr‐MPN)@RGD) to create a microenvironment that promotes cell colonization and immune regulation, improving the bone repair capability of orthopedic implants (**Scheme** **1**a). Our studies have shown that the coating can release Sr^2+^ in response to inflammatory microenvironments, mediating the immune response during the early stages of implantation to create an immune microenvironment conducive to bone repair. The introduction of RGD greatly improves the functional activities of surrounding BMSCs, such as adhesion, proliferation, differentiation, and polarization. The coating also exhibits long‐term regulation of osteoblast‐osteoclast differentiation, angiogenesis, and anti‐ROS activities, efficiently promoting the bone repair process of bone implants (Scheme 1b).

## 2 Experimental Section

### 2.1 Materials

Proanthocyanidin (PC, Cas: 4852‐22‐6), Iron (III) nitrate nonahydrate (Fe (NO_3_)_3_∙9H_2_O, Cas: 7782‐61‐8) and Strontium chloride hexahydrate (SrCl_2_∙6H_2_O, Cas: 10025‐70‐4) were obtained from Macklin. Sodium anhydrous acetate (Cas: 127‐09‐3) and tris (hydroxymethyl) aminomethane (Tris, Cas: 1185‐53‐1) were purchased from Aladdin. Polystyrene sphere (PS, 3 µm), NH_3_∙H_2_O, H_2_SO_4_, H_2_O_2_(30%), hydrogen chloride (HCl, 38%), sodium chloride (NaCl, 99.5%), sodium β‐glycerophosphate (β‐GP) and polypropylene (PP) tablets were purchased from Sigma. Cyclo(Arg‐Gly‐Asp‐D‐Phe‐Cys) (c(RGDfc)) acetate salt (c(RGDfc), 143P21) was purchased from TGpeptide Biotechnology (Nanjing, China). Phosphate buffered saline (PBS, P1010‐2L), 4′, 6‐diamino‐2‐phenylindole (DAPI, D8200), rhodamine phalloidin (Phalloidin‐TRITC, CA1610), BCIP/NBT alkaline phosphatase Kit (PR1100), and cell counting kit‐8 (CCK‐8, CA1210), paraformaldehyde (4%, P1110), Triton X‐100 (T8200) and alizarin red S solution (1%, pH4.2, G1452) were purchased from Solarbio Life Science. DMEM medium (PM150210), α‐MEM medium (PM150421), penicillin‐streptomycin solution (PB180120) and fetal bovine serum (164 210) were purchased from Pricella Life Science & Technology Co., Ltd. Serum‐free Cell cryopreservation solution (C40050) and universal antibody diluent (WB100D) were purchased from New Cell & Molecular Biotech. None of the chemicals were subjected to further purification. Cell culture flask (TCF011025), cell culture dish (TCP010006, TCP010024, and TCP010096), and PCR tubes (PCR520200) were purchased from Guangzhou Jet Bio‐Filtration Co., Ltd. Glass‐bottom cell culture plates (801 002 and 801 004) and frozen storage tube (612 521) were purchased from NEST Biotechnology. Taq Pro Universal SYBR qPCR Master Mix (Q712‐02) and HiScript III All‐in‐one RT SuperMix Perfect for qPCR (R333‐01) come from Vazyme. Silicon wafers (SSPs), quartz plates (Alfa Aesar) and glass covers (14 mm diameter) were washed with a mermaid solution (70% H_2_SO_4_ and 30% H_2_O_2_, V/V) at 98 °C for 2 h and rinsed with deionized (DI) water, Dry in a gentle stream of air before use (Note: piranha solution is highly oxidative and corrosive, and special care should be taken during preparation and use). The resistivity of the DI water used was greater than 18.25 MΩ cm after purification by the milli‐Q system.

### 2.2 Preparation of Coatings

#### 2.2.1 Preparation of MPN and (Sr‐MPN)@RGD Coatings

The preparation of MPN coating has been reported in published articles from our research group.^[^ ^28^, ^29^ ^]^ The preparation process of Sr‐MPN coating is shown in Scheme 1a. First, within a sodium acetate buffer system (pH = 4.5), Sr^2+^ at varying concentrations (6 mM) was mixed with PC solution (0.5 mM). Following this, Fe^3+^ (1.5 mM) was incorporated and the mixture was thoroughly mixed and then left undisturbed for 10 min. Finally, Tris‐HCl solution (pH = 8, 0.3 M) was added to stabilize the coating structure, and the coating was dried by gentle air flow. One layer of Sr‐MPN coatings was generated by completing these steps. By repeating this process “n” times, it is possible to achieve Sr‐MPN coatings with a thickness of “n” layers. “n” defaults to 5 unless specified otherwise.

#### 2.2.2 Preparation of (Sr‐MPN)@RGD Coating

The strategy of introducing c(RGDfc) molecules onto the surface of PC‐based MPN coatings has been reported in a previously published article.^[^ ^22^ ^]^ In brief, the fabrication process of (Sr‐MPN)@RGD coatings is shown in Scheme 1b. The prepared Sr‐MPN coatings were immersed in c(RGDfc) solution (pH = 8, 1 mg mL^−1^), sealed, and slowly shaken (20 rpm) for 24 h. Afterward, the coatings are rinsed with DI water to remove unbound c(RGDfc) molecules and thoroughly dried with a gentle stream of air, resulting in the formation of (Sr‐MPN)@RGD coatings.

### 2.3 Characterization

All prepared coatings were stored at 4 °C and dried thoroughly prior to characterization. The thickness of silicon wafer‐coated samples was determined by optical ellipsometry (M‐2000UI, J.A. Woollam). X‐ray photoelectron spectra of silicon wafer‐coated samples were obtained using a Thermo‐Electron ESCALAB 250 spectrometer with a monochromatic Al source (1486.6 eV). The water contact angles (WCA) of the Ti plate surface were measured by static sessile drop method on a KRUSS DSA1 v1.80 analyzer. Zeta potentials of hybrid coatings on polystyrene beads (Ø = 3 µm) were determined by Zetasizer Nano ZS ZEN3600 (WM2016002, Malvern). The surface morphology of the coatings was characterized using atomic force microscopy (AFM) with a Dimension Icon system (Bruker). The surface roughness was analyzed from the topographical maps by the subsidiary software. SEM was used to observe the surface morphology before and after coating on different shapes substrates, and EDS data were obtained. Coated and uncoated samples were coated with platinum using EM ACE600(60 s deposition, 20 mA). Microstructure was performed using a scanning electron microscope (WM2017015, HITACHI) at 3 kV.

#### 2.3.1 Factors of Sr‐MPN Coating Fabrication

Sr^2+^ concentration, deposition time per cycle, and different cycle numbers were explored. First, different concentrations (1.5/6/12 mM) of Sr^2+^ were mixed with 0.5 mM PC solution in buffer system (pH = 3/4.5/6). Subsequently, Fe^3+^ (1.0/1.5 mM) was added and the mixture was thoroughly mixed, then left undisturbed for 10 min, and the rest was unchanged.

#### 2.3.2 Stability of Sr‐MPN and (Sr‐MPN)@RGD Coatings

The Sr‐MPN and (Sr‐MPN)@RGD coating were immersed in DMEM medium for 0–30 days, and immersed in acidic buffer (pH = 3.5) and alkaline buffer (pH = 10.5). Subsequent to washing and drying, the residual thickness of the coating was measured by ellipsometer to evaluate the stability and degradation of the coating.

#### 2.3.3 Release Process of PC, Sr, and Fe in (Sr‐MPN)@RGD Coatings

The release of Fe and Sr from the (Sr‐MPN)@RGD coating in different pH was detected by Inductively Coupled Plasma‐Mass Spectrometry (ICP‐MS). Briefly, the (Sr‐MPN)@RGD coating was immersed in PBS (pH = 7.4) and acidic buffer (pH = 6.0), and the supernatants were collected intermittently over periods of 0–30 days and measured using ICP‐MS. The ion content in the supernatant was obtained after comparison with a standard curve prepared using Fe and Sr standards.

The release of PC from the (Sr‐MPN)@RGD coating was detected by UV‐Vis spectroscopy. Briefly, the (Sr‐MPN)@RGD coating was immersed in PBS (pH = 7.4) and acidic buffer (pH = 6.0), the supernatant was collected intermittently over 0–30 days periods, and the absorbance at 280 nm was measured using UV‐Vis. The PC content in the supernatant was obtained after comparison with a standard curve prepared using PC standards.

#### 2.3.4 Free Radical Scavenging Assays

The total antioxidant capacity of the coatings was assessed using the FRAP method. First, a working curve was established according to the protocol provided by the FRAP assay kit. FeSO_4_ solutions with specific concentrations (0, 0.15, 0.3, 0.6, 0.9, 1.2, and 1.5 mM) were prepared and added to a 96‐well plate, followed by the addition of the working solution (180 µL). After incubating for 5 min at 37 °C, the absorbance of the 96‐well plate was measured at 593 nm using a spectrophotometer. The total antioxidant activities of the coatings and Ti were determined immediately after preparation, following the same procedure. Based on the measured intensities, the antioxidant activity of each sample was calibrated using the standard curve.

The DPPH radical scavenging activity of the coatings was determined using the DPPH radical scavenging Capacity Assay Kit. Briefly, Ti and coatings (50 nm, 2 cm^2^) were mixed with the working solution. The absorbance at 515 nm was obtained using a Lambda 25 spectrophotometer. The calculation formula for the DPPH radical scavenging rate of the samples is as follows:

(1) $$\begin{array}{ccc} & & DPPH\;inhibition\;rate\;\;\left(\%\right)\\ & & =\left(Abs\;blank-\frac{Abs\;control-Abs\;sample}{Abs\;blank}\right)\;\times 100\% \end{array}$$

### 2.4 Cell Culture

The bone marrow macrophages (BMMs) and bone‐marrow mesenchymal stem cells (BMSCs) were isolated from the bone marrow of SPF Sprague Dawley rats aged 3 weeks as the previous study described.^[^ ^30^ ^]^ The human umbilical vein endothelial cells (HUVECs) obtained from Procell Life Science & Technology Co., Ltd. The BMSCs were planted on Cell Culture Bottle (75 mL volume) in α‐MEM medium supplemented with 100U mL^−1^ penicillin, 100 mg mL^−1^ streptomycin, and 10% FBS. The BMMs and HEUVC were cultured with DMEM medium supplemented with 100 U mL^−1^ penicillin, 100 U mL^−1^ streptomycin, and 10% FBS. All the cells were maintained in a humidified incubator with an atmosphere of 37 °C and 5% CO_2_. The medium was replaced every 2–3 days. The cells were passaged with 0.25% trypsin after reaching 90% confluence.

#### 2.4.1 Immune Responses on Different Coatings

##### Macrophage Polarization Analysis

BMMs were seeded onto different coatings and cultured in a pro‐inflammatory environment containing 50 ng mL^−1^ lipopolysaccharide (LPS, Sigma, USA) and 50 ng mL^−1^ interferon‐γ (IFN‐γ, Pepreotech, USA). After 48 hours, cells and supernatants were collected. First, Samples were fixed, blocked, and incubated overnight at 4 °C with fluorescein isothiocyanate (FITC) ‐labeled anti‐CD86 (M1 marker) antibody and Cyanine 3 (Cy3) ‐labeled anti‐CD206 antibody (M2 marker). After washing, the slides were sealed with an anti‐fluorescence quencher containing DAPI and imaged using LSCM. The area occupied by positive cells was analyzed by ImageJ. Second, polarized macrophage surface markers were analyzed by flow cytometry (FACS). Cells were incubated with fluorescein isothiocyanate (FITC)‐labeled anti‐CD86 antibody and allophycocyanin (APC)‐labeled anti‐CD206 antibody (Biolegend, USA) for 30 min at 4 °C to identify M1 (CD86^+^) and M2 (CD206^+^) phenotypes. Cell analysis was performed using a flow cytometer (CytoFLEX, Beckman Coulter, USA), and data were analyzed with CytExpert software.

Additionally, after seeding BMMs on different coatings and stimulating them with 50 ng mL^−1^ LPS and 50 ng mL^−1^ IFN‐γ, the supernatants of BMMs were evaluated for M1 (TNF‐α, IFN‐γ, IL‐1β, and IL‐6), M2 (IL‐4 and IL‐10) and other (TGF‐β, FGF, VEGF and RANKL) macrophage factors using enzyme‐linked immunosorbent assay (ELISA) kits (Multisciences, China) according to the manufacturer's instructions.

##### Immune Effects on Cells

The supernatants (CM) of BMMs cultured on Ti or MPN, Sr‐MPN, and (Sr‐MPN)@RGD coated Ti surfaces and add them to cells co‐incubated without coating to explore the effects of cytokines secreted by macrophages from different groups on the surrounding cells.

The cells were seeded onto the coated surfaces (5 × 10^4^ cells). Subsequently, the inflammatory macrophage culture supernatant (LI‐CM, the supernatant obtained after the BMMs were incubated with 50 ng mL^−1^ LPS and 50 ng mL^−1^ IFN‐γ for 48 hours) was added to mimic the inflammatory environment.

#### 2.4.2 BMSCs Migration Assay

Cell migration was analyzed using Transwell cell culture inserts (Corning, 8 µm, USA). First, BMMs were seeded on different coated surfaces (24‐well plate, 5 × 10^4^ cells), and then were induced in a proinflammatory environment with 50 ng mL^−1^ LPS and 50 ng mL^−1^ IFN‐γ, and the cell culture supernatant was collected 48 h later. Subsequently, the LPS and IFN‐γ induced culture supernatant (LI‐CM) of BMMs from different groups was added to the lower chamber (500 µL α‐MEM containing 0.1% FBS was added to each group except α‐MEM containing 10% FBS in the positive control group). The inner chamber of 8 µm Transwell was loaded with BMSCs cell suspension (2.5 × 10^5^ cells mL^−1^) in 200 µL serum‐free α‐MEM. After 24 hours of incubation, the cells were fixed with 4% paraformaldehyde for 15 min at room temperature. Non‐migrating cells on the upper surface of the membrane were removed with a cotton swab. The insert was washed with PBS, and the migrating cells at the bottom of the membrane were stained with 0.1% crystal violet for 5 min. Cell staining was observed and counted under an inverted microscope using ImageJ software.

#### 2.4.3 BMSCs Attachment and Spreading at Early Stage

BMSCs were seeded at a density of 4 × 10^4^ cells cm^−2^ on a 2 cm^2^ uncoated or coated surface with MPN, Sr‐MPN, or (Sr‐MPN)@RGD and cultivated with LI‐CM. At 2‐ and 4‐h post‐seeding, the slides were gently washed with PBS, followed by fixation with 4% paraformaldehyde for 15 min. Subsequently, the slides were treated with 0.1% Triton X‐100 in PBS for 10 min and then rinsed three times with PBS. All slides were stained with phalloidin‐TRITC (red) for actin filaments and with DAPI (blue) for nuclei visualization. Cell morphology was observed using a Laser Scanning Confocal Microscope (LSCM).

#### 2.4.4 Cell Viability Assay

Cell viability was conducted using the Cell Counting Kit‐8 (CCK‐8) according to the manufacturer's guidelines. Briefly, 5 × 10^4^ cells per well were seeded in a 24‐well plate on different coatings and cultivated with LI‐CM. At the end of the culture period, the medium was replaced with 350 µL of fresh medium containing 35 µL of CCK‐8 solution and incubated for an additional 2 h at 37 °C. Subsequently, the supernatant was transferred from the 24‐well plate to a 96‐well plate, and the absorbance at 450 nm for each well was measured using a multifunctional enzyme labeling instrument. Subsequently, BMSCs were stained using Calcein‐AM/PI Cell Viability Assay Kit, and the cell viability and proliferation were observed by LSCM after 3 days of culture on different coatings.

#### 2.4.5 Osteogenic Differentiation Assay

Osteogenic medium was prepared by adding 10 mM β‐glycerophosphate (Sigma‐Aldrich, USA), 0.1 µM dexamethasone (Sigma‐Aldrich, USA), and 0.25 mM ascorbic acid (Sigma‐Aldrich, USA) to α‐MEM medium containing 100 U mL^−1^ penicillin, 100 mg mL^−1^ streptomycin, and 10% FBS. BMSCs were cultured on different coatings with the osteogenic induction medium for several days to induce their differentiation into osteoblasts.

##### ALP Staining and ALP Activity Analysis

Alkaline phosphatase (ALP) staining and activity assay were performed as an early marker of osteogenic differentiation. BMSCs were seeded at 2 × 10^4^ cells per well. After 14 days of osteogenic induction, cells were stained using a BCIP/NBT alkaline phosphatase color development kit according to the manufacturer's instructions, observed, and imaged under a microscope to visualize ALP expression. In parallel, ALP activity was quantitatively assessed using an ALP activity assay kit.

##### ARS Staining Analysis

After 21 days of culture, BMSCs were fixed with 4% paraformaldehyde and stained with 0.1% alizarin red S solution (pH 4.2) for 30 min. Mineralized nodule formation was observed and imaged under a microscope. Calcium deposition was simultaneously extracted with 10% cetylpyridinium chloride for 1 h, and quantified by measuring the absorbance at 593 nm.

#### 2.4.6 Tube Formation Assay and Immunofluorescence Staining

To evaluate the angiogenic potential of HUVECs in vitro, a tube formation assay was conducted. Initially, HUVECs were plated at a density of 2 × 10^4^ cells per well in both uncoated and coated plates of a 24‐well plate and incubated for 48 hours. Subsequently, 50 µL of basement membrane matrix was added to each well of a 96‐well plate and allowed to solidify. The cells from each 24‐well plate were then centrifuged and plated onto the gel at a density of 5000 cells per well in serum‐free medium. After 6 h of incubation, tube‐like structures were visualized using an inverted microscope and quantified for length and number using ImageJ software.

HUVECs were plated at a density of 4 × 10^4^ cells per well in both uncoated and coated plates of a 24‐well plate. After 2 days of culture, the cells were fixed with 4% paraformaldehyde, washed with PBS, and permeabilized with 0.2% Triton X‐100 (Solarbio, China) for 15 min at room temperature. Subsequently, the cells were blocked with PBS containing 5% bovine serum albumin (BSA) for 1 h at room temperature to prevent non‐specific binding of CD31. Following blocking, the cells were incubated with a CD31 antibody overnight at 4 °C in the dark. After washing with PBS, the cells were stained with DAPI for 5 min to visualize the cell nuclei. Images were acquired using laser confocal microscopy, and the fluorescence intensity of CD31 was quantified using ImageJ software.

#### 2.4.7 Transcriptome Analysis of BMSCs

BMSCs were seeded on Ti and (Sr‐MPN)@RGD coated Ti and added with IL‐CM to mimic the inflammatory environment, and cultured for 7 days. Total RNA was extracted using the TRIzol reagent (Invitrogen, CA, USA) according to the manufacturer's protocol. RNA purity and quantification were evaluated using the NanoDrop 2000 spectrophotometer (Thermo Scientific, USA). RNA integrity was assessed using the Agilent 2100 Bioanalyzer (Agilent Technologies, Santa Clara, CA, USA). Then the libraries were constructed using VAHTS Universal V6 RNA‐seq Library Prep Kit according to the manufacturer's instructions. The transcriptome sequencing and analysis were conducted by OE Biotech Co., Ltd. (Shanghai, China). The libraries were sequenced on a llumina Novaseq 6000 platform and 150 bp paired‐end reads were generated. About 49 M raw reads for each sample were generated. Raw reads of fastq format were first processed using fastp and the low‐quality reads were removed to obtain the clean reads. Then about 47.4 M clean reads for each sample were retained for subsequent analyses. The clean reads were mapped to the reference genome using HISAT2. FPKM of each gene was calculated and the read counts of each gene were obtained by HTSeq‐count. PCA analysis was performed using R (v 3.2.0) to evaluate the biological duplication of samples.

Differential expression analysis was performed using the DESeq2. Q value < 0.05 and foldchange > 2 or foldchange < 0.5 was set as the threshold for significantly differential expression gene (DEGs). Hierarchical cluster analysis of DEGs was performed using R (v 3.2.0) to demonstrate the expression pattern of genes in different groups and samples. The radar map of top 30 genes was drawn to show the expression of up‐regulated or down‐regulated DEGs using R packet grader. Based on the hypergeometric distribution, GO, KEGG pathway, Reactome, and WikiPathways enrichment analysis of DEGs were performed to screen the significant enriched term using R (v 3.2.0), respectively. R (v 3.2.0) was used to draw the column diagram, the chord diagram, and bubble diagram of the significant enrichment term. Gene Set Enrichment Analysis (GSEA) was performed using GSEA software. The analysis was used a predefined gene set, and the genes were ranked according to the degree of differential expression in the two types of samples. Then it is tested whether the predefined gene set was enriched at the top or bottom of the ranking list.

#### 2.4.8 Intracellular ROS Level Assay

BMMs and BMSCs were seeded onto different coatings at a density of 5 × 10^4^ cells cm^−2^. After 24 h, the complete medium was replaced with medium containing 500 µM H_2_O_2_ and LI‐CM for 12 hours to induce oxidative stress. 2′,7′‐dichlorofluorescin diacetate (DCFH‐DA, S0033, Beyotime) was diluted to a final concentration of 10 µmol L^−1^ in serum‐free medium to detect ROS levels. The fluorescence intensity of the DCFH‐DA probe was imaged using a LSCM to visualize the spatial localization of ROS generation within cells.

#### 2.4.9 Osteoclast Differentiation Assay and TRAP Staining and TRAP Activity Analysis

Osteoclast induction medium was prepared by adding 50 ng mL^−1^ receptor activator of nuclear factor‐κB ligand (RANKL) (Pepreotech, USA) and 30 ng mL^−1^ macrophage colony‐stimulating factor (M‐CSF) (Pepreotech, USA) to DMEM medium containing 100 U mL^−1^ penicillin, 100 mg mL^−1^ streptomycin, and 10% FBS. BMMs were cultured in the osteoclast induction medium with LI‐CM for 5 days to induce their differentiation into osteoclasts.

Tartrate‐resistant acid phosphatase (TRAP) activity was detected using a TRAP Assay kit (Beyotime, China) according to the manufacturer's protocol. Briefly, samples were combined with an acid phosphatase buffer and incubated at 37 °C for 60 min, after which the reaction was stopped by adding 0.5 N NaOH. The absorbance was measured at 405 nm using a microplate reader to quantify the pNP produced from the substrate. TRAP activity was expressed as micromoles of pNP produced per minute per milligram of total cellular protein.

#### 2.4.10 Gene Expression Detected by RT‐qPCR

After cell culture, total RNA was extracted from the cultured cells using TRIzol Reagent. The concentration and purity of the extracted RNA were determined by measuring the absorbance at 260 and 280 nm. Equal amounts of RNA were then reverse transcribed into cDNA using the HiScript III All‐in‐one RT SuperMix Perfect for qPCR kit (Vazyme, China). Following reverse transcription, quantitative real‐time PCR (qRT‐PCR) was performed using a real‐time PCR system (LightCycler480, Roche, USA). The relative expression levels of target genes were normalized to the housekeeping gene β‐actin. Data were analyzed using the 2^−ΔΔCT^ method. The mRNA expression levels of inflammatory factors CD86, iNOS, CD206, IL‐1β, IL‐6, TGF‐β, and IL‐10, osteoclast‐related markers cathepsin K (CTSK) and tartrate‐resistant acid phosphatase (TRAP), as well as osteoblast‐related markers collagen type I (Col‐1), osteopontin (OPN), osteocalcin (OCN), and bone sialoprotein (ON) were evaluated. The primer sequences for the selected genes are listed in Table S1 (Supporting Information).

### 2.5 ELISA

BMMs were seeded in 24‐well plates with different coatings, after which the medium was replaced with serum‐free DMEM medium 24 hours after the addition of 50 ng mL^−1^ LPS and 50 ng mL^−1^ IFN‐γ. After continued culture for 48 h, cytokines in the supernatant were measured using ELISA kits according to the manufacturer's method (IFN‐γ, TNF‐α, IL‐1β, IL‐6, IL‐4, IL‐10, TGF‐β, FGF, VEGF, and RANKL).

### 2.6 Animal Test

Eighteen 6‐week‐old male Sprague Dawley (SD) rats (weighing: 300 g) were randomly divided into 4 groups (n = 3): Control group (uncoated titanium rods), Ti‐MPN group (titanium rods coated with MPN), Sr‐MPN group (titanium rods coated with Sr‐MPN), and Ti‐(Sr‐MPN)@RGD group (titanium rods coated with (Sr‐MPN)@RGD). After anesthesia with 10% chloral hydrate (1 mL per 100 g) via intraperitoneal injection, the skin on the lateral aspect of the femur was incised to expose the femoral bone. Under constant cooling with 0.9% NaCl solution, a 1 mm diameter and 5 mm deep hole was drilled perpendicular to the femur using a dental drill. The implants were then inserted into the femoral defects. The surgical site was disinfected with povidone‐iodine and sutured closed. Cefuroxime sodium was administered intramuscularly daily to prevent postoperative infection. At 4 and 8 weeks, the rats were euthanized with an overdose of chloral hydrate, and the femoral specimens were collected. The samples were fixed in 4% paraformaldehyde for further analysis. All experimental procedures were conducted in accordance with the Animal Care Guidelines of the Wenzhou Institute of the Chinese Academy of Sciences and approved by the Animal Ethics Committee of the Wenzhou Institute, with permission number WIUCAS23061302.

#### 2.6.1 Microfocus Computed Tomography (Micro‐CT)

The femora containing the implants were scanned using micro‐computed tomography (micro‐CT) at 80 kV, 300 µA, with a 360° rotation step of 0.6°. A region of interest (ROI) within 200 µm from the implant surface was reconstructed using the associated CTAn and CTVol software. Bone volume (BV), total volume (TV), and trabecular number (Tb. N) were measured from the 3D reconstructed images.

#### 2.6.2 Masson and H&E Staining

Masson's trichrome and hematoxylin and eosin (H&E) staining were performed according to previously described methods for evaluating bone formation. Briefly, for Masson's trichrome staining, deparaffinized and rehydrated femoral tissue sections were treated with Weigert's iron hematoxylin solution for 15 min. The slides were rinsed in running tap water for 5 min. Subsequently, the sections were stained with picro‐sirius red solution for 6 min, differentiated in phosphomolybdic acid solution, and counterstained with 1% acetic acid solution. After coverslipping, each stained slide was imaged under a light microscope to assess bone regeneration. For H&E staining, the slides were first deparaffinized and rehydrated, followed by staining with hematoxylin solution for 10 min. The slides were then rinsed in running tap water, treated with 80% ethanol for 2 min, and stained with eosin solution for 5 min. After coverslipping, each stained slide was imaged under a light microscope to evaluate bone regeneration.

#### 2.6.3 Immunohistochemical Analysis

The completely decalcified femoral tissue was embedded in paraffin and sectioned into 5 µm slices using a microtome (Leica, RM2255). The sections were deparaffinized in xylene and rehydrated through graded ethanol solutions. The sections were then permeabilized in 0.3% Triton‐X 100 in PBS for 10 min and blocked with 0.04 g mL^−1^ bovine serum albumin (BSA, Sigma) in PBS for 1 h. The tissues were stained with fluorescein isothiocyanate (FITC)‐conjugated anti‐CD86 (CD86, 1:100) and Cyanine 3 (Cy3)‐conjugated anti‐CD206 (1:100) antibodies to confirm M1 and M2 macrophage distribution, respectively. Then the tissues were subsequently stained with FITC‐conjugated anti‐VEGF (VEGF, 1:100) and Cy3‐conjugated anti‐OCN (OCN, 1:100) antibodies to confirm blood vessel formation and bone formation. Cell nuclei were counterstained with 4′,6‐diamidino‐2‐phenylindole (DAPI, 1:1000). The CD86‐, CD206‐, VEGF‐ and OCN‐positive areas were quantified using ImageJ software. The positive areas were quantified as the ratio of the CD86‐, CD206‐, VEGF‐, or OCN‐positive area to the total cell expression area.

### 2.7 Data Processing and Statistical Analysis

Statistical analyses were performed using GraphPad Prism 9.5 (GraphPad Software Inc., CA, USA), and graphical representations were generated using Origin 2023b (OriginLab, MA, USA). The results are expressed as mean ± standard deviation (SD). The significance between experimental groups was assessed using Student's t‐test and indicated by the symbols *, **, and *** for *p* < 0.05, *p* < 0.01, and *p* < 0.001, respectively. Additionally, statistical significance compared to the Control group or POS‐control group was marked by ^#^ *p* < 0.05, ^##^ *p* < 0.01, or ^###^ *p* < 0.001, while non‐significant differences were labeled as “ns.” All experiments were independently replicated three times, unless otherwise specified.

## 3 Results

### 3.1 Characterization of Strontium Incorporation

**Figure 1:**
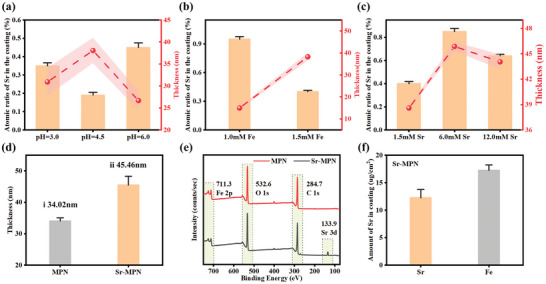
Characterization of strontium incorporation. a) Setting the molar concentration of Fe and Sr both set to 1.5 mM, under different pH levels, XPS analysis showed that the proportion of Sr element (yellow histogram) and the elliptic polarization instrument measured coating thickness (red line). b) At a fixed pH of 4.5 and a Sr molar concentration of 1.5 mM, as the molar concentration of Fe was varied, the XPS‐detected Sr element composition (yellow histogram) and thickness of coatings (red line) were measured. c) With a pH of 4.5 and a fixed Fe molar concentration of 1.5 mM, as different molar concentrations of Sr were introduced, the XPS‐detected Sr element composition (yellow histogram) and thickness of coatings (red line) were measured. d) The thickness of MPN and Sr‐MPN coating. e) XPS wide spectra scan of MPN and Sr‐MPN coating. f) Total amount of Sr and Fe at Sr‐MPN coating detected by ICP‐MS. N = 3.

The Sr‐MPN can be coated on the surface of the substrate by immersing the substrate in a PC solution and a Fe^3+^ solution containing Sr at a controlled pH value. Prior to proceeding, it is essential to determine the optimal thickness of the Sr‐MPN and the ideal content of incorporated Sr. The thickness of the Sr‐MPN coating and the amount of incorporated Sr were optimized by varying the pH value, the concentration of Sr^2+^ and Fe^3+^ added, with the objective of obtaining the thickest coating and the highest content of incorporated Sr simultaneously. The differences were reflected by the thickness of the coatings utilizing ellipsometry and the Sr content by X‐ray Photoelectron Spectroscopy (XPS). Recognizing that pH is a pivotal factor in the formation of MPNs. First, the pH effect of coating thickness and Sr^2+^ content was investigated by fixing the concentration of Sr^2+^ and 1.5 mM Fe^3+^ and adjusting the pH of the buffer solution at 3.0, 4.5, and 6.0. As depicted in **Figure** **1**a, a pH of 4.5 yielded the thickest Sr‐MPN coating, albeit with a relatively low Sr composition. Subsequently, fixing the pH at 4.5, fixing the molar concentration of Sr^2+^ at 1.5 mM, exploring the changes in coating thickness and Sr^2+^ content when the molar concentration of Fe ion was 1.0, 1.5, and 6.0 mM, respectively. Figure 1b indicates that a concentration of 1.5 mM Fe^3+^ resulted in the thickest coating, although with limited Sr enrichment. Lastly, while maintaining a pH = 4.5 and 1.5 mM Fe^3+^, the influence of Sr molar concentration on the coating thickness and the Sr content was tested. It is interesting to note that Sr concentration at 6 mM concurrently enhanced both coating thickness and the Sr content (Figure 1c), However, a further increase in the concentration of Sr^2+^ does not lead to a thicker coating thickness. This may be due, to the ionic strength of SrCl_2_ as a salt solution that drives MPN construction, as pointed out in previous studies.^[^ ^31^ ^]^ The increased ion concentration shields the phenolic hydroxyl groups in the polyphenols, moving them away from the Fe III center and interacting with other complexes in solution to form thicker and rougher membranes. However, the further enhancement of the ion concentration will only make the film rougher rather than thicker.^[^ ^31^ ^]^ Based on the above results, the conditions (1.5 mM Fe, 6 mM Sr, and buffer pH = 4.5) were selected as the optimal to construct Sr‐MPN coating. As shown in Figure 1d, the average thickness of the optimized Sr‐MPN coating amounted to ≈45.46 ± 0.83 nm, surpassing the MPN coating (34.02 nm) prepared at the same conditions without Sr. XPS spectra of coating also proved the presence of Sr element, with an approximate atomic percentage of 0.85% (Figure 1e). Upon complete digestion of a Sr‐MPN coating (≈44 nm thick) using nitric acid, inductively coupled plasma mass spectrometry (ICP‐MS) was used to quantitatively determine the Fe and Sr content in the Sr‐MPN coating. As shown in Figure 1f, after conversion using a standard curve (Figure S1, Supporting Information), the Sr‐MPN coating prepared using the standard method contained ≈17.23 ± 0.99 µg cm^−2^ of total Fe and 12.24±1.51 µg cm^−2^ of total Sr. Table S2 (Supporting Information) present the elemental distribution ratios determined by XPS, respectively.

### 3.2 Characterization of (Sr‐MPN)@RGD Coating

**Figure 2:**
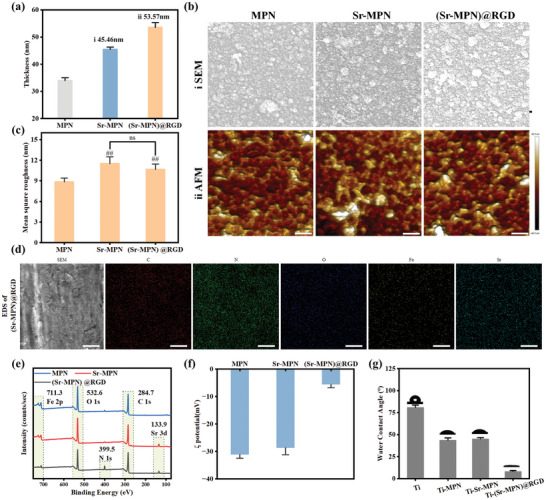
Characterization of MPN, Sr‐MPN, and (Sr‐MPN)@RGD coatings. a) The thickness of the different coatings was measured by elliptography. b) Representative (i) SEM and (ii) frontal AFM image of the different coatings synthesized on the surface of Ti slices. c) Mean square roughness (Rq) of the different coatings. d) Representative EDS of (Sr‐MPN)@RGD coating. e) XPS wide spectral scan of the different coatings. f) Average zeta potential of different coatings on the surface of the PS pellets. g) Water contact angle of different coatings on Ti plate. The scale bars in (b–i) are 100 nm, (b–ii) are 250 nm, and (d) are 50 nm. Using t‐test in (c), no significance noted as “ns,” *p < 0.05, **p < 0.01, ***p < 0.001 compared between the two group, and #*p* < 0.05, ##*p* < 0.01 or ###*p* < 0.001 compared with the MPN group. N = 3.

We fixed c(RGDfc) molecules through a click reaction with PC and fixed them on the surface of the Sr‐MPN coating as reported in the previous paper,^[^ ^22^ ^]^ resulting in a (Sr‐MPN)@RGD coating with a thickness of 53.57±1.82 nm (**Figure** **2**a ii). Compared to the original Sr‐MPN (Figure 2a i), ≈8.11 nm increased for (Sr‐MPN)@RGD, indicating the successful deposition of RGD. The surface morphology and element distribution of MPN, Sr‐MPN, and (Sr‐MPN)@RGD coatings were analyzed using scanning electron microscopy (SEM). As shown in Figure 2b i, all coatings exhibited a uniformly distributed granular and rough surface. Atomic force microscopy (AFM) was utilized to investigate the nanoscale roughness of the MPN, Sr‐MPN, and (Sr‐MPN)@RGD coated surfaces (Figure 2b ii). As shown in Figure 2c, the mean square roughness (Rq) of MPN and Sr‐MPN coatings was increased compared to bare substrates. However, compared to Sr‐MPN coating, the Rq of the coated surface did not change much after c(RGDfc) grafting, which was similar to the results of our previous study.^[^ ^22^ ^]^ We probed the elemental distribution on the (Sr‐MPN)@RGD coated surface using energy dispersive spectroscopy (EDS) in Figure 2d. The presence of uniformly distributed Sr and N elements was detected on the surfaces of (Sr‐MPN)@RGD coatings, directly verifying the successful introduction of Sr and c(RGDfc) into MPN.

The characterization studies were conducted to assess the changes in surface properties of the coated substrates. XPS was further employed to investigate the elemental composition of the coatings. The survey spectra (Figure 2e) revealed that the (Sr‐MPN)@RGD coatings exhibited Sr and N signals, which successfully demonstrated the successful immobilization of c(RGDfc), which could be confirmed by the detailed split‐peaks of C1s, Sr3d, and Fe2p (Figure S2, Supporting Information). The surface potential of the coatings applied to polystyrene (PS) microspheres with a diameter of ≈3 µm was measured using a nanoparticle size analyzer. As depicted in Figure 2f, MPN and Sr‐MPN exhibited similar surface potentials of ≈−30 mV. However, the surface potential of the coating after RGD grafting increased significantly (ζ potential = −5.68 ± 1.17 mV), indicating the successful grafting of RGD. The wettability of the coatings applied to Ti plates was investigated using a water contact angle (WCA) meter. As shown in Figure 2g, the original Ti surface exhibited a WCA of 80.95° ± 2.6°. Compared to the bare Ti substrate, the Ti surfaces coated with MPN, Sr‐MPN, and (Sr‐MPN)@RGD exhibited significantly increased hydrophilicity, with the (Sr‐MPN)@RGD coating demonstrating the highest wettability (WCA = 8.16° ± 0.98°). This can be attributed to the hydrophilic properties of PC and c(RGDfc) molecules, as well as the increased nanoscale roughness of (Sr‐MPN)@RGD coatings. Previous studies have suggested that changes in interfacial morphology and properties can improve the osteogenic microenvironment.^[^ ^32^ ^]^ On one hand, a rough surface favors the formation of blood clots and the activation of platelets, while reducing the number of inflammatory cells.^[^ ^33^ ^]^ This further influences the cellular mechanisms involved in faster and improved osseointegration. On the other hand, hydrophilic surfaces have been shown to had significant potential in promoting the initial adhesion and subsequent biological behavior of osteoblasts and immune cells.^[^ ^34^ ^]^

### 3.3 Stability and Release Behavior of (Sr‐MPN)@RGD Coating

**Figure 3:**
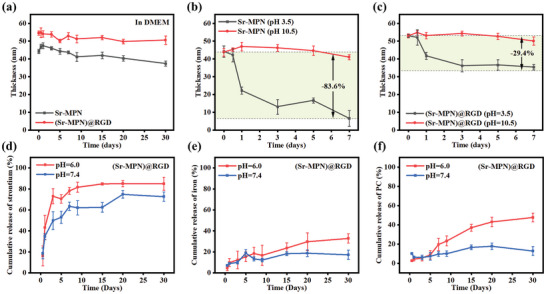
Analysis of thickness change of Sr‐MPN and (Sr‐MPN)@RGD coating in a) DMEM, b) pH = 3.5, and c) pH = 10.5 at 37 °C. Release of d) Sr^2+^, e) Fe^3+^ and f) PC from (Sr‐MPN)@RGD coating in different pH at 37 °C. N = 3.

As a composite coating with the potential to promote tissue integration and repair, it is imperative to maintain stability under physiological conditions. To assess this, the Sr‐MPN and (Sr‐MPN)@RGD coating was immersed in Dulbecco's Modified Eagle Medium (DMEM) (pH = 7.4), and its thickness was monitored over time (**Figure** **3**a). The results indicated that there was slightly decrease in the thickness of Sr‐MPN and (Sr‐MPN)@RGD coating during the 30‐day observation period, which indicated that these coatings could exist stably under physiological conditions. Furthermore, the degradation behavior of the coating was investigated by monitoring the change of the coating thickness under different pH conditions. The Sr‐MPN and (Sr‐MPN)@RGD coating were immersed in buffer solutions with pH values of 3.5 and 10.5, and its thickness was monitored over time (Figure 3b,c). The results showed that there was no significant decrease in thickness of Sr‐MPN and (Sr‐MPN)@RGD coating in the buffer solution with pH 10.5 over the 7‐day observation period, indicating the stability of the coating in alkaline environments. However, in the buffer solution with pH 3.5, a more significant decrease in coating thickness was observed in Sr‐MPN coating, suggesting that the coating is susceptible to degradation and component release under acidic conditions. However, the (Sr‐MPN)@RGD coating demonstrates a better ability to resist acidic conditions, evidenced by less reduction in coating thickness, which has also been confirmed in our previous studies.^[^ ^35^ ^]^ These results suggested that both in physiological conditions and acidic or alkaline conditions, the stability of the coating of the RGD composite coating is better than that of Sr‐MPN coating.

Upon the occurrence of peri‐implantitis, the pH of the local tissue microenvironment decreases due to the production and accumulation of various chemical substances and acidic metabolites. To further characterize the release behavior of the newly prepared coating within the inflammatory microenvironment. The release of Sr and Fe ions, as well as PC, from the (Sr‐MPN)@RGD coating was measured using inductively coupled plasma mass spectrometry (ICP‐MS) and UV‐visible absorption spectroscopy, respectively, under physiological conditions (pH = 7.4) and in a simulated inflammatory environment (pH = 6.0). Under physiological conditions (pH = 7.4), a rapid release of Sr^2+^ was observed within the first 7 days, followed by a slow but steady release after 7 days, ultimately reaching a maximum concentration (Figure 3d i). Furthermore, the release behavior of Sr^2+^ changed significantly under the simulated inflammatory conditions (pH = 6.0), exhibiting a markedly accelerated release rate (Figure 3d ii). This suggests that the (Sr‐MPN)@RGD coating exhibits inflammatory responsiveness, enabling a more timely and effective release of Sr^2+^ to modulate the immune microenvironment during inflammation. The release profiles of Fe^3^⁺ and PC from (Sr‐MPN)@RGD exhibited trends similar to those observed for Sr^2^⁺ (Figure 3e,f). The fast release kinetic at pH 6 is primarily attributed to the pH‐responsive nature of the super molecular interactions in MPN.^[^ ^26^ ^]^ However, the total cumulative releases of Fe^3^⁺ and PC were significantly lower compared to Sr^2^⁺, which may be attributed to the weaker interaction between Sr^2^⁺ and polyphenols.^[^ ^36^ ^]^ On the other hand, the substantial proportion of residual PC and Fe indicates that the (Sr‐MPN)@RGD structure remains largely intact during the Sr^2^⁺ release process. These results indicate that Fe and PC, as the primary constituents of the coating structure under the aegis of the c(RGDfc), endow the coating with excellent stability. Whereas Sr exists as a releasable component whose release does not compromise the stability of the newly designed coating. The standard curves used for these measurements are shown in Figures S2 and S3 (Supporting Information).

Additionally, due to the catechol‐containing PC, which serves as a hydrogen bond acceptor and donor with a polyhydroxy structure, our coating containing PC can interact with various substances and adhere to a wide range of complex‐shaped substrates (Figure S4, Supporting Information). This versatility and adaptability contribute to the coating's potential for widespread applications in tissue engineering and regenerative medicine.

### 3.4 (Sr‐MPN)@RGD Coating Regulated Macrophage Polarization and Cytokine Secretion In Vitro

**Figure 4:**
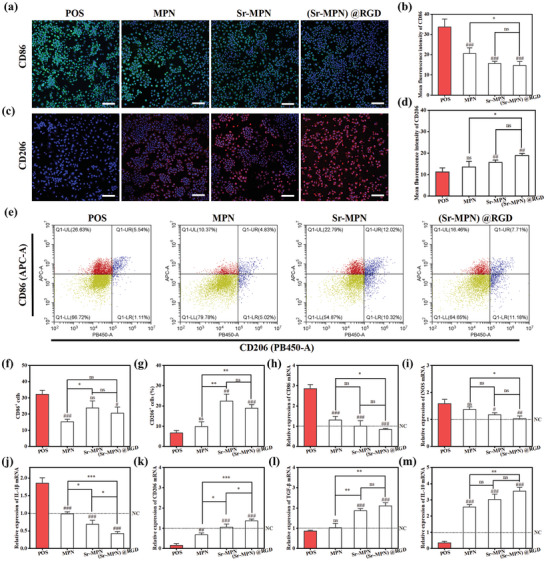
MPN, Sr/ MPN, and (Sr‐MPN)@RGD coatings regulated macrophage polarization in vitro. Representative images of a) CD86 (M1, green) and b) CD206 (M2, red) immunofluorescence staining of cells cultured on Ti, Ti‐MPN, Ti‐Sr‐MPN, and Ti‐(Sr‐MPN)@RGD coated surfaces after stimulation with LPS and IFN‐γ for 48 h. e) Flow cytometric analysis demonstrating M1 (CD86, APC) and M2 (CD206, PB450) phenotypic polarization of macrophages on various coatings. Quantitative analysis presented the proportion of M1 (CD86^+^) f) and M2 (CD206^+^) g) macrophages. qPCR analysis of the relative mRNA expression of h) CD86, i) iNOS, j) IL‐1β, k) IL‐10, l) TGF‐β and m) CD206 in cells cultured on different coated surfaces after LPS and IFN‐γ stimulation for 48 h. The dashed line in (h‐m) represents the negative control group. Scale bars in (a) and (c) are 50 µm. N = 3, using t‐test, no significance noted as “ns,” *p < 0.05, **p < 0.01, ***p < 0.001 compared between the two group, and #*p* < 0.05, ##*p* < 0.01 or ###*p* < 0.001 compared with the Positive Control group.

Macrophage polarization dominates the early immune response. The effects of MPN, Sr‐MPN, and (Sr‐MPN)@RGD coatings on macrophage infiltration and phenotypic transformation (M1 & M2) were investigated. BMMs were cultured on MPN, Sr‐MPN, and (Sr‐MPN)@RGD coated surface and stimulated with LPS and IFN‐γ for 48 h for subsequent analysis. The group treated with LPS and IFN‐γ only was referred to as the POS‐control group. First, the cells on the coating were analyzed by cellular immunofluorescence imaging, the number of infiltrating CD86‐positive (M1 maker, green fluorescent) macrophages were reduced in all coating groups compared to positive controls, with the most significant reductions in the Sr‐MPN and (Sr‐MPN)@RGD coating groups (**Figure** **4**a,b). The number of CD206 (M2 maker, red fluorescent) positive macrophages on the Sr‐MPN and (Sr‐MPN)@RGD coated surfaces was significantly increased after 48 hours of co‐culture (Figure 4c,d). Induced macrophages were further analyzed by flow cytometry using PB450‐labelled CD206 antibody and APC‐labelled CD86 antibody. After 48 h of co‐culture, there was a decrease in the percentage of infiltrating CD86‐positive (M1‐labelled) macrophages and an increase in the percentage of CD206‐positive (M2‐labelled) macrophages in all the coated groups, with a significant increase in the CD206‐positive cells on the surface of the Sr‐MPN and (Sr‐MPN)@RGD coatings as compared to the Sr‐neutral group (Figure 4e–g). The relative expression levels of M1‐marked genes such as CD86, iNOS, and IL‐1β were significantly down‐regulated on MPN, Sr‐MPN, and (Sr‐MPN)@RGD coatings by qPCR analysis of mRNA extracted from the corresponding macrophages, with (Sr‐MPN)@RGD coating being the most pronounced (Figure 4h–j). The expression levels of M2 marker genes such as IL‐10, TGF‐β, and CD206 were slightly up‐regulated in the Sr‐MPN and (Sr‐MPN)@RGD‐coated groups (Figure 4k–m), which was consistent with the immunofluorescence and flow cytometric results. The above experimental results showed that the newly constructed Sr‐MPN and (Sr‐MPN)@RGD coatings exhibited excellent ability to promote macrophage M2 polarization and inhibit M1 polarization. More delicately, polarization ground inhibition of M1 macrophages may be mainly influenced by PC and Fe in the composite coating, whereas polarization of M2 macrophages is dominated by Sr^2+^ release.

Macrophages have a significant impact on cell recruitment and modulation of the osteogenic microenvironment through paracrine cytokines.^[^ ^37^ ^]^ Therefore, the cytokine content contained in the supernatants of inflammation‐induced macrophages cultured on different coated surfaces for 48 h were tested by enzyme‐linked immunosorbent assay (ELISA). Figure S5a‐d (Supporting Information) confirmed the decrease of IFN‐γ, TNF‐α, IL‐1β, and IL‐6 (pro‐inflammatory cytokines) in all coatings containing PC. Figure S5e, f (Supporting Information) confirmed that the increase of IL‐4 and IL‐10 (anti‐inflammatory cytokines) in MPN, Sr‐MPN, and (Sr‐MPN)@RGD‐coated groups, with the more significant changes in Sr‐MPN and (Sr‐MPN)@RGD‐coated group than MPN coating. And, ELISA testing also revealed a rise in cytokines associated with the promotion of tissue healing and angiogenesis (TGF‐β, FGF, and VEGF) in the (Sr‐MPN)@RGD‐coated group, as well as a rise in cytokines that promoted osteoclast differentiation (RANKL) in the positive control group and a decrease in the (Sr‐MPN)@RGD‐coated group (Figure S5g‐j, Supporting Information). The data gathered clearly demonstrated that (Sr‐MPN)@RGD coatings possessed notable anti‐inflammatory and pro‐healing properties. The presence of PC significantly suppressed the expression of M1 phenotypic markers, mainly attributed to its anti‐inflammatory properties,^[^ ^23^ ^]^ and the presence of Sr significantly increased the expression of M2 phenotypic markers and prompted macrophage polarization toward the M2 phenotype.^[^ ^11^ ^]^ These paracrine cytokines are known to have a significant effect on the behavior of surrounding cells.^[^ ^11^ ^]^ Hence, the (Sr‐MPN)@RGD coating, which targets bone marrow macrophages, is likely to significantly impact surrounding cells through cytokine modulation, thereby facilitating bone repair. (Figure S5k, Supporting Information).

### 3.5 (Sr‐MPN)@RGD Coating Regulated Proliferation, Migration, and Adhesion of BMSCs

In the subsequent experiment, two distinct types of experiments were conducted. One experiment involved extracting the supernatants (CM) of macrophages cultured on different coated surfaces and adding them to cells co‐incubated without coating. This was done to explore the effects of cytokines secreted by macrophages from different groups on the surrounding cells. The other experiment involved adding the inflammatory supernatants of LPS‐ and IFN‐γ‐stimulated macrophages (LI‐CM) to all treatment groups. This was done to explore the response of cells cultured on different coatings to adverse environments.

**Figure 5:**
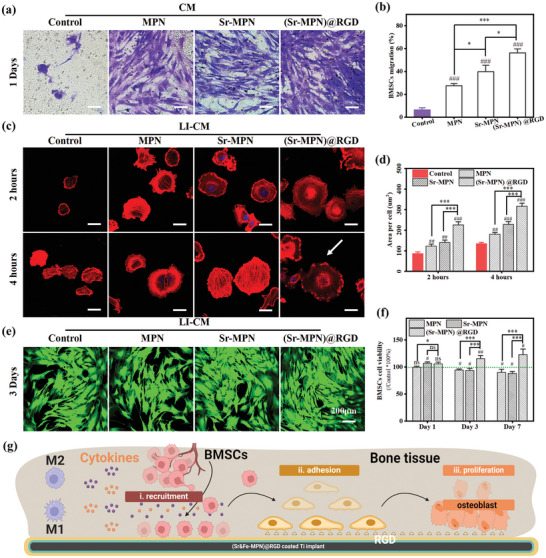
Cell attachment, proliferation, and migration on MPN, Sr‐MPN, and (Sr‐MPN)@RGD coatings. a) Optical image of BMSCs migration. b) The number of migrating BMSCs cells obtained by statistical analysis from (e). c) BMSCs cultured on different coatings for 2 and 4 h were stained with nuclear (blue) and phalloidin (red) fluorescence. d) The area of each cell was statistically analyzed from (c). e) Calcein‐PI staining of BMSCs cultured on different coatings for 3 days. f) CCK8 assay of BMSCs cultured on different coatings at various time points. g) (i) Schematic representation of the coating regulating macrophages to secrete a variety of cytokines to recruit BMSCs, and the composite coating promoting BMSCs (ii) adhesion and (iii) proliferation. Scale bars in (a) are 100 µm, in (c) are 50 µm, in (e) are 200 µm. N = 3, using t‐test, no significance noted as “ns,” *p < 0.05, **p < 0.01, ***p < 0.001 compared between the two group, and #*p* < 0.05, ##*p* < 0.01 or ###*p* < 0.001 compared with the Control group.

In the process of bone regeneration, the recruitment and mobilization of endogenous stem cells play a crucial role in the bone reconstruction phase. The effects of cytokines secreted by macrophages cultured with different coatings on the recruitment of BMSCs were observed using Transwell migration assays. Different groups of supernatants (CM) were placed in the lower chamber (medium containing 0.1% FBS and CM), and BMSCs were planted in the upper chamber of the cells (medium without FBS) to observe the migration of BMSCs. The number of cells (blue‐violet) on the bottom surface of the upper chamber of the Transwell in the Sr‐MPN and (Sr‐MPN)@RGD groups was significantly more than that on the surface of the MPN group at the same migration time (**Figure** **5**a,b). The above results suggest that cytokines secreted by macrophages cultured on the surface of the coating group have good pro‐cell migration ability and can recruit BMSCs to aggregate to the damaged bone localities.

After being recruited to the bone implant site, enhanced and earlier cellular adhesion by BMSCs proved pivotal in facilitating their localized engraftment. The colonization ability of stem cells on different coated surfaces under inflammatory conditions (with the addition of LI‐CM) was further verified by early adhesion experiments. Cell nuclei were stained with 4′, 6‐diamidino‐2‐phenylindole (DAPI, blue) and the cytoskeleton was stained with rhodamine‐labeled phalloidin (TRITC‐Phalloidin, red), and the cell adhesion to the coated surfaces of the BMSCs was observed after 2 and 4 h of incubation. Fluorescence images showed that BMSCs were uniformly distributed on all surfaces (Figure 5c), with the largest cell spreading area on the (Sr‐MPN)@RGD surface, and more densely packed F‐actin filamentous structures (red lines) as well as abundant finger‐like protrusions and filamentous pseudopods were observed (indicated by white arrows). Calculation of the average area of individual cells showed that at the adherent cell level, (Sr‐MPN)@RGD exhibited the most significant increase in cell spreading area compared to MPN and Sr‐MPN coatings (Figure 5d). The above results may be attributed to the removal of unfavorable ROS and inflammatory factors by the coating, allowing the cells to exhibit better adhesion to the coating. Meanwhile, the presence of RGD on the surface of the coating, which acts as a recognition site for integrins, exhibited excellent pro‐cell adhesion in this coating. Finally, BMSCs were cultured on the coating and subjected to live/dead staining and CCK‐8 assays to assess the cytocompatibility of each sample. Live/dead staining results are shown in Figure 5e, with live cells fluorescing green and dead cells red. Almost no dead cells were observed from all the coated surfaces, indicating good cytocompatibility of all the coatings. The time‐dependent proliferation of BMSCs on coated surfaces was evaluated using the CCK‐8 assay. The viability of cells cultured on Sr‐MPN and MPN coated surfaces showed inhibition at 3 and 7 days compared to the control (dashed line), suggesting that both surfaces are not conducive to long‐term cell colonization (Figure 5f). However, the cell viability of cells cultured on the (Sr‐MPN)@RGD coated surface was stronger than that of the control group, and more significantly better than that of the Sr‐MPN and MPN coated surfaces, both at 1, 3, and 7 days, which suggests that the RGDs show good pro‐proliferative activity for BMSCs in the short and long term.

These results indicated that the (Sr‐MPN)@RGD coating played multiple beneficial roles in the migration of BMSCs around bone implants. First, the (Sr‐MPN)@RGD coating modulated the polarization state of macrophages through PC and Sr^2+^, enhancing the chemotactic migration of BMSCs (Figure 5g–i). Second, the RGD in the (Sr‐MPN)@RGD coating better supported the initial adhesion of BMSCs on the implant surface (Figure 5g–ii), while BMSCs that adhered well to the coating can survive and proliferate effectively (Figure 5g–iii). In summary, the synergy of PC, Sr^2+^, and RGD can provide long‐term beneficial effects for bone regeneration and osseointegration.^[^ ^38^ ^]^

### 3.6 (Sr‐MPN)@RGD Coating Promoted Cell Osteogenesis and Angiogenesis In Vitro

**Figure 6:**
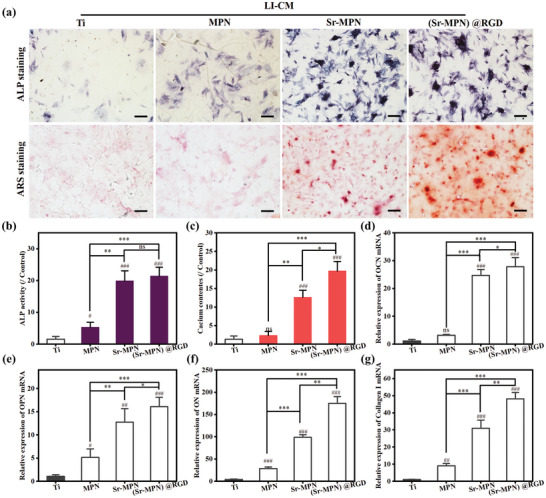
Osteodifferentiation of BMSCs on different modified substrates. a) Representative images of ALP staining after 7 days of co‐culture with coatings with LI‐CM and Alizarin Red S staining after 21 days. b) Quantitative analysis of ALP activity. c) Quantitative analysis of calcium deposition. Relative mRNA expression of d) OCN, e) OPN, f) ON, and g) College I genes in BMSCs cultured in different modified substrates for 7 days. Scale bars in (a) are 200 µm. N = 3, using t‐test, no significance noted as “ns,” *p < 0.05, **p < 0.01, ***p < 0.001 compared between the two group, and #*p* < 0.05, ##*p* < 0.01 or ###*p* < 0.001 compared with the Control group.

The higher osteogenic activity of osteoblasts during the bone healing process favors new bone formation and thus promotes the integration of the implant into the bone.^[^ ^39^, ^40^ ^]^ We investigated the in vitro osteogenic differentiation of BMSCs cells in inflammatory conditions (in LI‐CM) on different coated surfaces. After 7 days of osteogenic induction, ALP staining (**Figure** **6**a) and activity analysis (Figure 6b) showed that Sr‐MPN and (Sr‐MPN)@RGD coatings supplemented with Sr significantly enhanced early osteogenesis compared to MPN, with BMSCs cultured on (Sr‐MPN)@RGD coatings exhibiting the most pronounced osteogenic activity. ARS staining (Figure 6a) and calcium quantification (Figure 6c) of BMSCs cultured in osteogenic induction medium for 21 days also showed that Sr‐MPN and (Sr‐MPN)@RGD‐coated surfaces showed stronger mineralization of bone formation relative to MPN surfaces. Thus, we can clearly observe that Sr is one of the key factors promoting osteogenic differentiation of BMSCs, while RGD also shows a role in promoting osteogenesis.

Cellular osteogenic differentiation is a complex process and RT‐qPCR was further used to assess the expression of osteogenesis‐related genes (OCN, OPN, ON, and Coll‐I) in BMSCs cells cultured for 7 days on different coating surfaces (Figure 6d‐h). The results showed that Sr‐MPN and (Sr‐MPN)@RGD coatings significantly up‐regulated the mRNA levels of OCN, OPN, ON, and Coll‐I in surface‐cultured BMSCs, which were markedly different from those of MPN coating. And the ON and Collagen I levels of BMSCs cultured on the (Sr‐MPN)@RGD coating were obviously different from those of the Sr‐MPN coating without RGD. Overall, the newly designed (Sr‐MPN)@RGD coating material effectively up‐regulated the expression of osteogenesis‐related genes in BMSCs cells, and significantly promoted the osteogenesis and mineralization of bone formation of BMSCs under inflammatory conditions.

The angiogenic potential of endothelial cells, following a 6‐hour incubation in CM with various coatings, was assessed through an angiogenesis assay. As depicted in Figure S6a (Supporting Information), vascularization was evident after 6 hours of cellular growth. In both the control and MPN groups, the presence of scattered cell clusters without discernible tubular structures was observed. Conversely, the Sr‐MPN group exhibited only a limited number of incomplete tubular branches, whereas the (Sr‐MPN)@RGD group displayed abundant and well‐defined tubular structures. Statistical analysis, presented in Figure S6b‐c (Supporting Information), revealed significant variations in the number of nodes and junctions containing RGD and Sr materials compared to the control group. Notably, the (Sr‐MPN)@RGD group demonstrated the most pronounced effect, followed by the Sr‐MPN group. To further investigate the expression of angiogenesis‐related factors on the coating surface, immunofluorescence staining for the endothelial adhesion molecule CD31 was conducted, as shown in Figure S6d (Supporting Information). The (Sr‐MPN)@RGD group exhibited robust green fluorescence surrounding the cells, indicative of high CD31 expression. Statistical analysis of CD31 immunofluorescence, presented in Figure S6e (Supporting Information), revealed significant differences between the (Sr‐MPN)@RGD and control groups, with the control group's expression being slightly lower than that of the Sr‐MPN and MPN groups. Subsequently, the gene expression levels of VEGF and CD31 were quantitatively analyzed in Figure S6f‐g (Supporting Information). The (Sr‐MPN)@RGD group demonstrated the highest expression levels for both factors.

### 3.7 RNA‐Seq‐Based Transcriptome Analysis of the BMSCs Cultured on (Sr‐MPN)@RGD Coating

Bone marrow mesenchymal stem cells (BMSCs) were osteogenic induced on Ti‐ and Ti‐(Sr‐MPN)@RGD coating for 7 days in culture supernatants (LI‐CM) from inflammation‐stimulated BMMs. Total RNA was extracted for absolute quantitative transcriptome sequencing to explore the molecular mechanisms underlying the effects of (Sr‐MPN)@RGD coating on the biological processes of surface‐grown BMSCs and their ability to act, and screened for differential gene expression analysis. Pearson's correlation value and Principal Component Analysis (PCA) showed that the Ti group (Sr‐MPN)@RGD group met the quality control criteria and there was a significant difference between the groups (Figure S7a,b, Supporting Information), this suggests that (Sr‐MPN)@RGD significantly affects gene expression in cells. Using DeSeq2 package to find differentially expressed genes (DEGs). The volcano diagram showed that there were 815 up‐regulated genes and 729 down‐regulated genes in Ti group Vs Ti‐ (Sr‐MPN)@ RGD group (Figure S8, Supporting Information). The radar map and heat map of the first 50 changed genes are shown in Figure S9a, b (Supporting Information). It could be observed that genes related to the development and functional maintenance of cartilage and bone, such as Acan, genes related to maintaining the normal structure and function of blood vessels, such as Eln, genes related to extracellular matrix remodeling, such as F13a1, and genes related to cell growth and differentiation, such as lgfbp2, are upregulated. Additionally, downregulation can be observed in the MMP3 gene, which is involved in regulating the release of inflammatory mediators and the infiltration of inflammatory cells, and the Nox1 gene, which is involved in the production and regulation of ROS.

**Figure 7:**
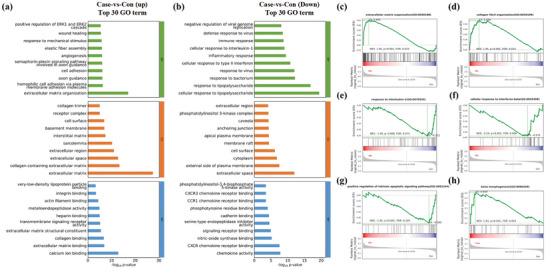
Transcriptome sequencing analysis of BMSCs cultured on Ti and Ti‐(Sr‐MPN)@RGD coating after osteogenic induction for 7 days. GO pathway enrichment analysis of DEGs with a) up and b) down expression. GSEA analysis of the regulation of c) extracellular matrix organization, d) collagen fibril organization, e) response to interleukin‐1, f) cellular response to interferon‐beta, g) positive regulation of intrinsic apoptotic signaling pathway, and h) bone morphogenesis.

Subsequently, Gene Ontology (GO) and Kyoto Encyclopedia of Genes and Genomes (KEGG) enrichment analyses were conducted on the differentially expressed genes (DEGs) of the Ti group and the Ti‐(Sr‐MPN)@RGD group. Gene ontology (GO) enrichment analysis showed that in the DEGs Top 30 enrichment analysis of Ti Vs Ti‐(Sr‐MPN)@RGD, DEGs was found to be upregulated in biological processes such as extracellular matrix organization, cell adhesion, angiogenesis, and wound healing, and downregulated in a variety of inflammatory responses (cellular response to lipopolysaccharide and cellular response to interleukin‐l, etc.). In the cellular component, it was mainly manifested as the influence on the cell membrane and extracellular matrix. Enrichment in molecular functions such as integrin binding, collagen binding, and calcium ion binding has also been identified (**Figure** **7**a,b). Additionally, Genome Set Enrichment Analysis (GSEA) also demonstrated that in the Ti‐(Sr‐MPN)@RGD group, genes associated with cell adhesion, such as extracellular matrix organization and collagen fibril organization, were significantly upregulated (Figure 7c,d)). Genes related to anti‐inflammatory responses, including response to interleukin‐1 and cellular response to interferon‐beta, (Figure 7e,f), as well as genes involved in anti‐apoptosis, specifically the positive regulation of the intrinsic apoptotic signaling pathway, were downregulated (Figure 7g), while gene promoting bone morphogenesis (Figure 7h) was significantly upregulated.

KEGG pathway analysis showed that the upregulated DEGs were involved in pathways mainly enriched in signaling pathways such as Focal adhesion, ECM‐receptor interaction, cAMP signaling pathway, cGMP‐PKG signaling pathway, Calcium signaling pathway, Complement and coagulation cascades and Vascular smooth muscle contraction signaling pathway (Figure S10a, Supporting Information). These enrichments suggest that (Sr‐MPN)@RGD coating can modulate the osteogenic capacity of BMSCs through multiple pathways. The down‐regulated genes were mainly enriched in inflammation‐related pathways such as TNF and IL‐17 (Figure S10b, Supporting Information), suggesting that (Sr‐MPN)@RGD coating could modulate the excessive inflammatory response around the implant to improve the osteogenic microenvironment, which may be closely related to the release of PC and Sr. To validate the impact of the coating on BMSCs in an inflammatory environment, further investigations were conducted in subsequent experiments.

### 3.8 (Sr‐MPN)@RGD Coating Inhibited Radical Oxidative Stress and Osteoclast Differentiation In Vitro

Osteoporosis, fractures, and invasive surgical procedures for bone implants often lead to the generation and accumulation of a large amount of free radicals (such as O^2−^, ·OH, H_2_O_2_·, NO·, etc.). The subsequent radical oxidative stress (ROS) can exacerbate inflammatory responses and even lead to cell death, thereby affecting bone healing (Figure S11a, Supporting Information). A reasonably designed implant coating can confer good ROS resistance on the implant surface.^[^ ^41^ ^]^ First, the antioxidant capabilities of MPN, Sr‐MPN, and (Sr‐MPN)@RGD coatings were thoroughly characterized using FRAP and DPPH assay kits. As shown in Figure S11b‐c (Supporting Information), compared to the bare titanium plate group, there were significant changes in the color of DPPH and FRAP solutions after treatment with titanium plates modified with MPN, Sr‐MPN, and (Sr‐MPN)@RGD coatings. There were no significant differences in the free radical scavenging abilities among the three coating groups, all demonstrating good free radical scavenging capabilities. This may be attributed to the fact that the free radical scavenging ability of the coatings is positively correlated with the content of phenolic hydroxyl groups, and there was no significant difference in the amount of PC contained in the three coating groups. To further validate the intracellular ROS scavenging properties of MPN, Sr‐MPN, and (Sr‐MPN)@RGD coatings, a pathological microenvironment simulating oxidative stress was created on different coatings with hydrogen peroxide (H_2_O_2_) + LI‐CM, and intracellular ROS was labeled with the fluorescent probe 2′,7′‐dichlorodihydrofluorescein diacetates (DCFH‐DA). As shown in Figure S11d, f (Supporting Information), the levels of ROS in BMSCs and BMMs cells increased after treatment on the surface of bare titanium plates. In contrast, the average fluorescence intensity of BMSCs and BMMs cells on the surfaces of MPN, Sr‐MPN, and (Sr‐MPN)@RGD coatings were significantly reduced (Figure S10e,g, Supporting Information). These results indicate that the coatings containing PC attenuate the oxidative stress in BMSCs and BMMs in inflammatory environments, thereby improving the pathological microenvironment.

Osteoclasts derived from BMM affect bone remodeling in multiple directions. Both inflammation and ROS significantly affect osteoclast differentiation,^[^ ^42^, ^43^ ^]^ so we investigated the effect of different coatings on the differentiation of BMM into osteoclasts. First, BMM cells cultured on the coatings were treated with M‐CSF and RANKL, and after 5 days the BMMs formed osteoclasts with multinucleated structures. In contrast to the remarkable generation of osteoclasts on Ti plates, the majority of BMMs grown on MPN, Sr‐MPN, and (Sr‐MPN)@RGD coatings remained in a mononuclear morphology (Figure S12a, Supporting Information). The number of multinucleated cells in each image was counted, and the results of Figure S12b (Supporting Information) showed that Sr‐MPN and (Sr‐MPN)@RGD coatings more significantly reduced multinucleated cell formation. TRAP activity assay showed more than 3.5‐fold reduction in TRAP activity in BMMs cultured on (Sr‐MPN)@RGD coating compared to POS controls (Figure S12c, Supporting Information). In addition, we assessed the expression levels of osteoclast differentiation and inflammatory genes in BMMs grown on different coatings. MPN, Sr‐MPN, and (Sr‐MPN)@RGD coatings all significantly down‐regulated the expression of TRAP and CTSK genes compared to POS controls (Figure S12d,e, Supporting Information). We also found a decrease in the pro‐inflammatory marker IL‐6 and an increase in the anti‐inflammatory marker IL‐10 in all osteoblastic differentiation‐induced BMMs cultured with polyphenol‐containing coatings (Figure S12f,g, Supporting Information), and the changes in the (Sr‐MPN)@RGD‐coated group appeared to be the most definitive. The above findings suggest that (Sr‐MPN)@RGD coating can definitively inhibit osteoblastic differentiation during remodeling of injured bone.

### 3.9 (Sr‐MPN)@RGD Coating Augmented Osseointegration In Vivo

**Figure 8:**
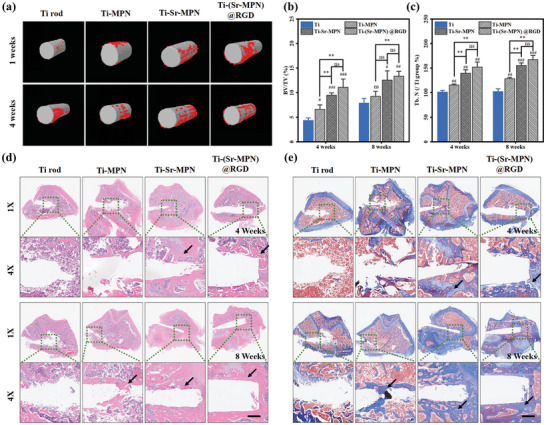
Osteoinductive and osseointegration capacities of functionalized Ti‐based materials in vivo. a) Micro‐CT was used to detect the quality of the regenerated bone around the implanted Ti rods containing different coatings after 4 and 8 weeks. b) Comparison of the bone volume fraction (BV/TV %) of the implants of (a). c) Comparison of the trabecular number (Tb. N 1 mm^−1^) of the implants of (b). d) Representative HE staining images of bone tissue (BT) around different implants in epiphysis regions. e) Representative Masson staining images of new bone maturity. The blue and red represent low and high maturity bone tissue, respectively. Scale bars in (d,e) are 200 µm. N = 3, no significance noted as “ns,” *p < 0.05, **p < 0.01, ***p < 0.001 compared between the two group, and #*p* < 0.05, ##*p* < 0.01 or ###*p* < 0.001 compared with the Control group, using t‐test.

To further investigate the effect of (Sr‐MPN)@RGD coating on in vivo osseointegration in rats, an implantation study of Ti implants modified in the external epicondyle of the rat femur was performed. The density of the surrounding bone was assessed using micro‐X‐ray computed tomography (Micro‐CT) 4 and 8 weeks after implantation. Representative bone CT reconstruction images as shown in **Figure** **8**a around the implant (blue pseudo‐color) showed varying degrees of bone regeneration (white pseudo‐color), where the bone density around the (Sr‐MPN)@RGD was significantly higher than that in the Ti rob group and the MPN group. Quantitative analysis at weeks 4 and 8 showed that the percentage of bone volume (bone volume/tissue volume, BV/TV) was significantly higher in the (Sr‐MPN)@RGD group than in the Ti group, which implies an increase in the number of bone trabeculae, and greater bone anabolism than catabolism (Figure 8b). Concurrently, the (Sr‐MPN)@RGD coating significantly increased trabecular thickness (Tb. Th), indicating a transition from parallel‐fibered bone to lamellar bone deposition (Figure 8c).

We used HE and Masson trichrome staining to further analyze the formation of new bone around different implants. H&E staining images (Figure 8d) showed the formation of thicker new bone tissue (black arrows) around the Sr‐doped implants (Sr‐MPN and (Sr‐MPN)@RGD coatings, especially the latter). The contact area of the Sr‐MPN and (Sr‐MPN)@RGD coatings with the new bone was also significantly increased compared to the control group. Subsequently, similar results were observed in Masson's trichrome staining images (Figure 8e). More new bone formation (indicated by black arrows) could be observed in the Sr‐MPN and (Sr‐MPN)@RGD coating groups. As the color of Masson's trichrome staining gradually changes with the maturation of bone (from blue to red), we conclude that the new bone formation in the (Sr‐MPN)@RGD coating group is more mature compared to that in the Sr‐MPN coating group. Overall, the (Sr‐MPN)@RGD coating not only promotes the formation of new bone but also has a positive impact on its maturation.

### 3.10 (Sr‐MPN)@RGD Coating Augmented Inflammation and Angiogenesis In Vivo

**Figure 9:**
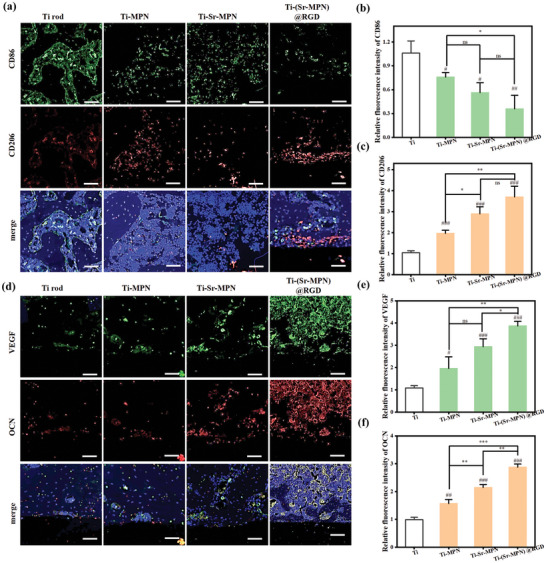
Inflammation, angiogenesis, and osteogenesis of bone tissue around Ti rods coated with (Sr‐MPN)@RGD coating. a) Representative fluorescent images showing the expression of CD86 (M1 marker, green), and CD206 (M2 marker, red) at the bone‐implant interface 4 weeks after implantation. Fluorescence quantification of b) CD86 and c) CD206 in (a). d) Representative fluorescent images showing the expression of VEGF (green), and OCN (red) at the bone‐implant 8 weeks after implantation. Fluorescence quantification of e) VEGF and f) OCN in (b). Scale bars in (a) and (d) are 100 µm. N = 3, no significance noted as “ns,” *p < 0.05, **p < 0.01, ***p < 0.001 compared between the two group, and #*p* < 0.05, ##*p* < 0.01 or ###*p* < 0.001 compared with the Ti rod group, using t‐test.

To further investigate the impact of the (Sr‐MPN)@RGD coating on immune modulation and angiogenesis/osteogenesis in the tissues surrounding the implants in rats, we performed immunofluorescence staining for CD86, CD206, VEGF, and OCN on tissue sections. The immunofluorescence staining and its quantification related to inflammation revealed the presence of chronic inflammation around the tissues surrounding the Ti rod in the femoral condyle of rats, manifesting as downregulation of CD86 and upregulation of CD206 expression (**Figure** **9**a–c). However, the coating groups significantly downregulated the expression of CD86, with the Sr‐containing coatings exhibiting a remarkable ability to upregulate CD206 expression. Overall, these data suggest that the (Sr‐MPN)@RGD coating confers anti‐inflammatory properties to the implant and has great potential to modulate the desired regenerative bone immune microenvironment, where M2 macrophages produce growth factors to enhance the differentiation of mesenchymal progenitor cells and further promote bone healing. Further examination of the expression of angiogenesis‐related (VEGF) and osteogenesis‐related (OCN) proteins in the surrounding tissues at 8 weeks after implantation revealed significant findings (Figure 9d–e). In particular, endothelial cells expressing VEGF were visibly present in the tissues surrounding the Ti rods coated with the (Sr‐MPN)@RGD coating, indicating that the composite coating containing Sr and RGD significantly promoted angiogenesis, which is beneficial for subsequent osteogenesis. In addition, Figure 9d and Figure 9f shows that the (Sr‐MPN)@RGD coating group exhibited the most significant expression of OCN compared to the uncoated Ti rods and other coating groups.

## 4 Discussion

The diversification of orthopedic implants has achieved significant success.^[^ ^44^ ^]^ However, the issue of poor osseointegration on the surface of bone implants remains, often leading to failed implantations.^[^ ^45^ ^]^ Such failures often necessitate the removal of implants and subsequent reoperations, undoubtedly increasing patient suffering and societal burden.^[^ ^46^ ^]^ In previous works, investigators have primarily focused on constructing coatings with antibacterial, antioxidative,^[^ ^47^ ^]^ anti‐inflammatory,^[^ ^48^ ^]^ and osteogenic properties^[^ ^21^, ^49^ ^]^ on the surface of bone implants to enhance their success rate, which has also achieved remarkable success. Among them, recent research has gradually begun to focus on the immunomodulatory effects of implants and suggests that a comprehensive immunomodulatory system, rather than a single cell or simple system. Liu et al. constructed a multifunctional MPN nanocoating containing Sr to achieve early immunomodulation on bone implants.^[^ ^48^ ^]^ Wang et al. designed a bone implant surface coating with two metals that modulates the inflammatory environment around the implant into an anti‐inflammatory immune environment.^[^ ^50^ ^]^ On the other hand, for early bone damage remodeling on the surface of bone implants, promoting stem cell adhesion and colonization is one of the key factors for successful osseointegration. As Ding et al. suggested, polydopamine (PDA) coatings led to a decrease in cell viability in standard tests, which was attributed to energy consumption caused by enhanced local cell motility.^[^ ^51^ ^]^ Our previous research also indicated that modifying the MPN coating surface with c(RGDfc) can effectively enhance its osseointegration ability mainly by enhancing early cell adhesion.^[^ ^22^ ^]^ Therefore, we believe that more comprehensive materials should simultaneously consider two challenges: promoting stem cell colonization and immunomodulatory capacity.

Our research proposes a (Sr‐MPN)@RGD coating with potent stem cell colonization and immunomodulatory capabilities and demonstrates its significant bone regeneration and osseointegration abilities. First, our experimental results demonstrate that the newly constructed (Sr‐MPN)@RGD coating can adhere to the surface of various implants, co‐assemble Sr^2+^and RGD molecules on PC/Fe‐MPN coating, and retain the biological activity of these components. Our experimental results also demonstrate that the Sr content in the Sr ‐MPN coating can be adjusted by pH, Sr^2+^, and Fe^3+^ input concentration. The grafting of c(RGDfc) leads to changes in the surface properties of the coating, further enhancing the stability of the rest of the coating structure in acidic environments, achieving slow release of Sr^2+^ in the inflammatory microenvironment around the implant while ensuring the retention of other coating components without disintegration.

Further research reveals the significant potential of the (Sr‐MPN)@RGD coating during the early stages of damaged bone repair. First, under inflammatory acidic conditions, the substantial and efficient release of Sr in the early stages regulates the immune response, facilitating a smooth transition of macrophages from the M1 phenotype to the M2 phenotype and modulating their paracrine cytokine secretion. This process significantly influences the subsequent recruitment and establishment of BMSCs, as well as maintaining a balance between osteogenic and osteoclastic activities on the coating surface. Second, BMSCs play a direct role in the bone repair mechanism, and their attachment, proliferation, and subsequent differentiation on the implant surface are crucial determinants of successful osteointegration.^[^ ^52^ ^]^ The (Sr‐MPN)@RGD coating containing RGD significantly promotes early stem cell colonization and accelerates subsequent biological events, which, together with bone immune regulation, determine the outcome of bone regeneration. Finally, our results also show that (Sr‐MPN)@RGD coating can significantly scavenge ROS in adverse environments, promote osteogenesis to inhibit osteoblasts, and ultimately demonstrate excellent promotion of bone regeneration and bone‐implant integration in rat femurs. In addition, most implants used in clinical practice are irregularly shaped and have complex properties. In contrast, our simple implant modification method is capable of coating the surface of implants with complex shapes in a wide range of materials. Therefore, the application of an (Sr‐MPN)@RGD implant coating with stem cell colonization and immunomodulatory effects has been comprehensively demonstrated both in vitro and in vivo.

## 5 Conclusion

In conclusion, a bioactive coating (Sr‐MPN)@RGD was developed on various substrates in a simple and efficient manner. This coating exhibits excellent biocompatibility and antioxidant properties and is capable of converting adherent macrophages from the M1 phenotype to the M2 phenotype, thereby effectively inhibiting inflammatory responses. Furthermore, the (Sr‐MPN)@RGD composite coating has been observed to significantly promote stem cell recruitment and colonization, accelerate osteogenic differentiation, inhibit osteoblastic differentiation, and enhance neovascularization in inflammatory environments. In vivo experiments have demonstrated that the (Sr‐MPN)@RGD composite coating can enhance osseointegration in a more efficacious and expeditious manner, which may prove valuable for clinical application. The present study offers a novel design concept for addressing the intricate biological mechanisms involved in osseointegration of implants.

## Conflict of Interest

The authors declare no conflict of interest.

## Author Contributions

C.W. and Z.S. contributed equally to this work. C.W., Z.S., Q.W., and X.Z. investigated, designed the study, and wrote the original manuscript. C.W., Z.S., and C.X. performed most experiments. W.L. and H.L. conducted AFM‐related tests. K.H., Z.S., and C.X. analyzed data with intellectual contributions. X.Z. and Q.W. revised the manuscript. X.Z. and L.L. supervised this work and acquired the funding. All authors have read and approved the final manuscript for submission.

## Supporting information



Supporting Information

## Data Availability

The data that support the findings of this study are available on request from the corresponding author. The data are not publicly available due to privacy or ethical restrictions.
