# Supplementary material for: Enhancing the Implant Osteointegration via Supramolecular Co‐Assembly Coating with Early Immunomodulation and Cell Colonization
Source: Adv Sci (Weinh). 2025 Jan 13;12(9):2410595. doi: 10.1002/advs.202410595 (PMC11884616; doi:10.1002/advs.202410595)
Supplement: Supplementary file 1 — Supporting Information [file ADVS-12-2410595-s001.docx]

**Enhancing the Implant Osteointegration via Supramolecular Co-assembly Coating with Early Immunomodulation and Cell Colonization**

*Chenglong Wang,^¶ #^* *Zeyu Shou,^ф, § #^ Chengwei Xu,^§^ Kaiyuan Huo,^♀^ Wenjie Liu,^♀, Ѱ^ Hao Liu,^Ѱ^ Xingjie Zan ^♀^ *, Qing Wang^Ƥ^ *, and Lianxin Li^¶^ **

*^¶^* Department of Orthopaedics Surgery, Shandong Provincial Hospital Affiliated to Shandong First Medical University, Jinan, 250021, Shandong, China

*^ф^* Department of Orthopedics, Zhuji People's Hospital of Zhejiang Province, Zhuji Affiliated Hospital of Wenzhou Medical University, Shaoxing, Zhejiang, 311800, China

*^§^* Department of Orthopedics, The First Affiliated Hospital of Wenzhou Medical University, Wenzhou, Zhejiang, 325000, China

*^♀^* Wenzhou Institute, University of Chinese Academy of Sciences, Wenzhou Key Laboratory of Perioperative Medicine, Wenzhou, Zhejiang, 325001, China

*^Ѱ^* School of Materials Science and Engineering, Zhengzhou University, Zhengzhou 450001, China

*^Ƥ^* Yongkang First People's Hospital of Wenzhou Medical University, Jinhua, 321300, China

*^#^* Equal contribution author

*Corresponding author. E-mail: zanxj@ucas.ac.cn (X. Zan); [wangqinglijun@163.com](mailto:wangqinglijun@163.com) (Q. Wang); 13505312449@163.com (L. Li);

**
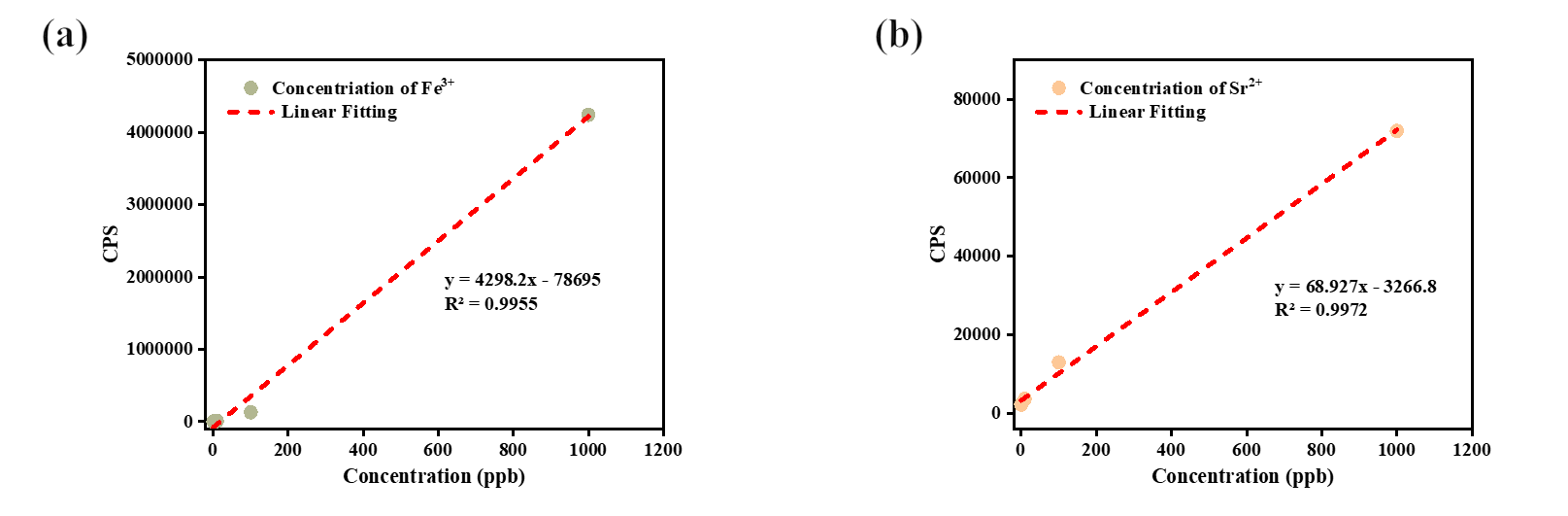
Figure S1.** Standard curves for (**a**) Fe and (**b**) Sr during ICP-MS testing.


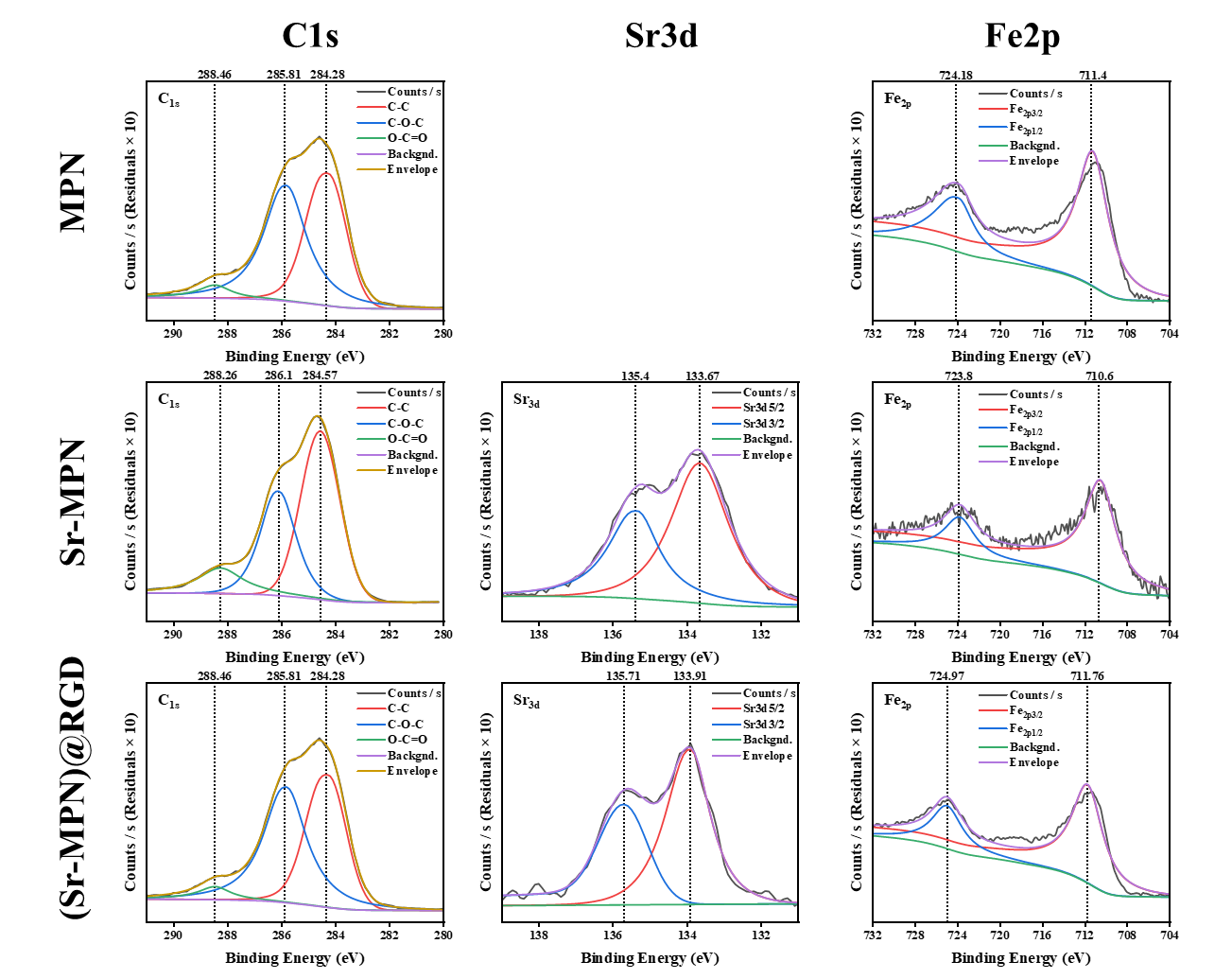
**Figure S2.** The split peaks of C1s, Sr3d and Fe2p in MPN, Sr-MPN and (Sr-MPN)@RGD.


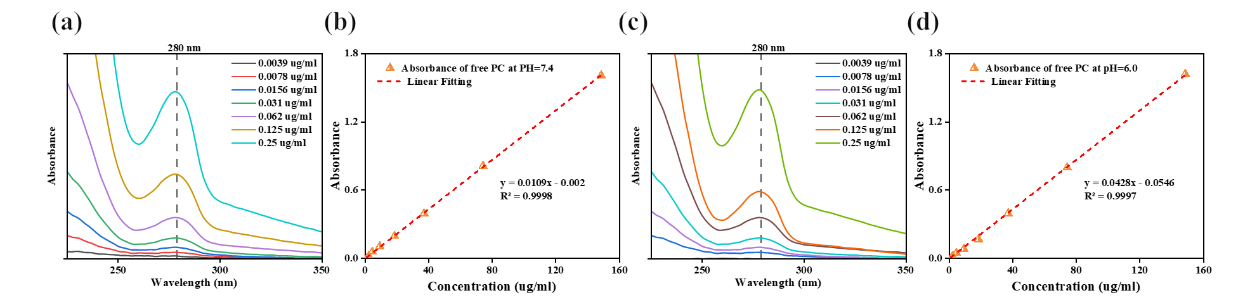
**Figure S3.** UV absorption spectra of PC solution with different concentrations at (a) pH=7.4 and (c) pH=6.0. Concentration-UV absorbance standard curves for PC at (b) pH=7.4 and (d) pH=6.0.


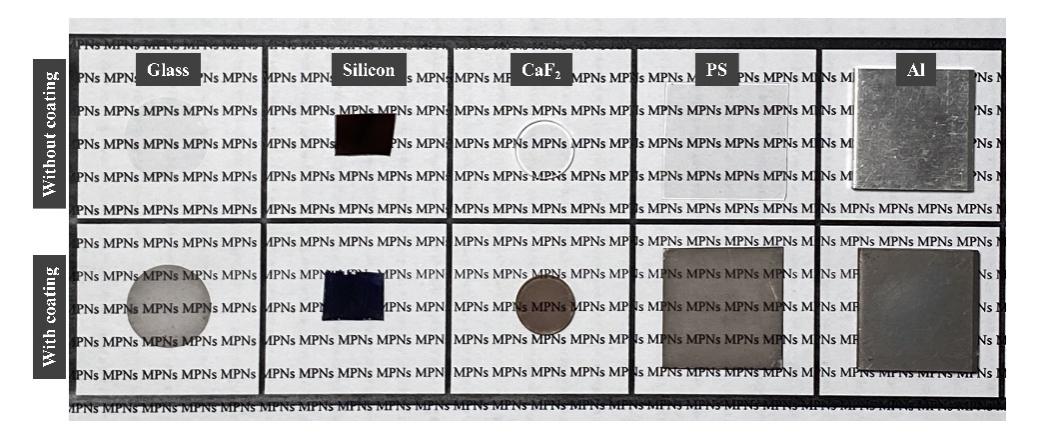
**Figure S4**. Photographs of glass sheets, silicon sheets, calcium fluoride, PS plastic sheets and Al surfaces with or without PC-based MPN coating.


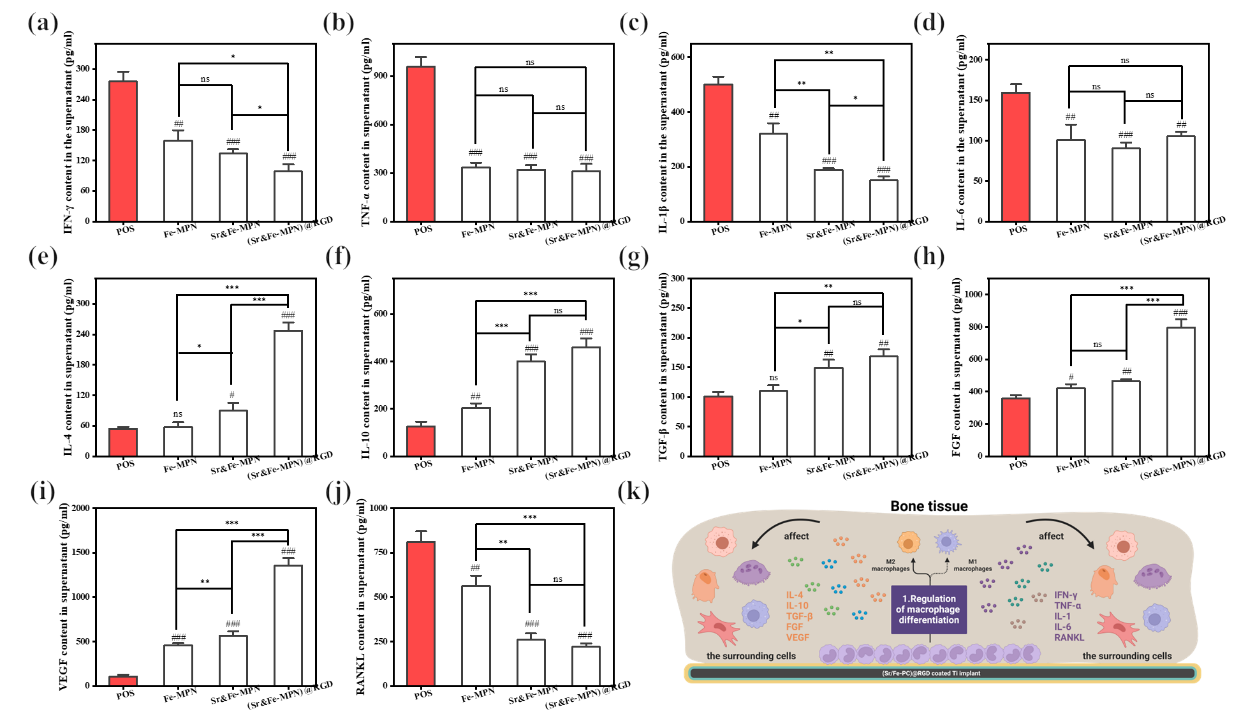
**Figure S5**. Fe-MPN, Sr&Fe-MPN and (Sr&Fe-MPN)@RGD coatings affected the secretion of cytokines by macrophages. The concentration of (**a**) IFN-γ, (**b**) TNF-α, (**c**) IL-1β, (**d**) IL-6, (**e**) IL-4, (**f**) IL-10, (**g**) TGF-β, (**h**) FGF, (**i**) VEGF and (**j**) RANKL at 48 hours secreted by BMM cells stimulation analyzed by ELISA. **(k)** Schematic representation of the coating regulating the secretion of a variety of cytokines by macrophages and its effects on surrounding cells. N=3, using t-test, no significance noted as "ns," *p < 0.05, **p < 0.01, ***p < 0.001 compared between the two group, and #p < 0.05, ##p < 0.01 or ###p < 0.001 compared with the Control group.

**
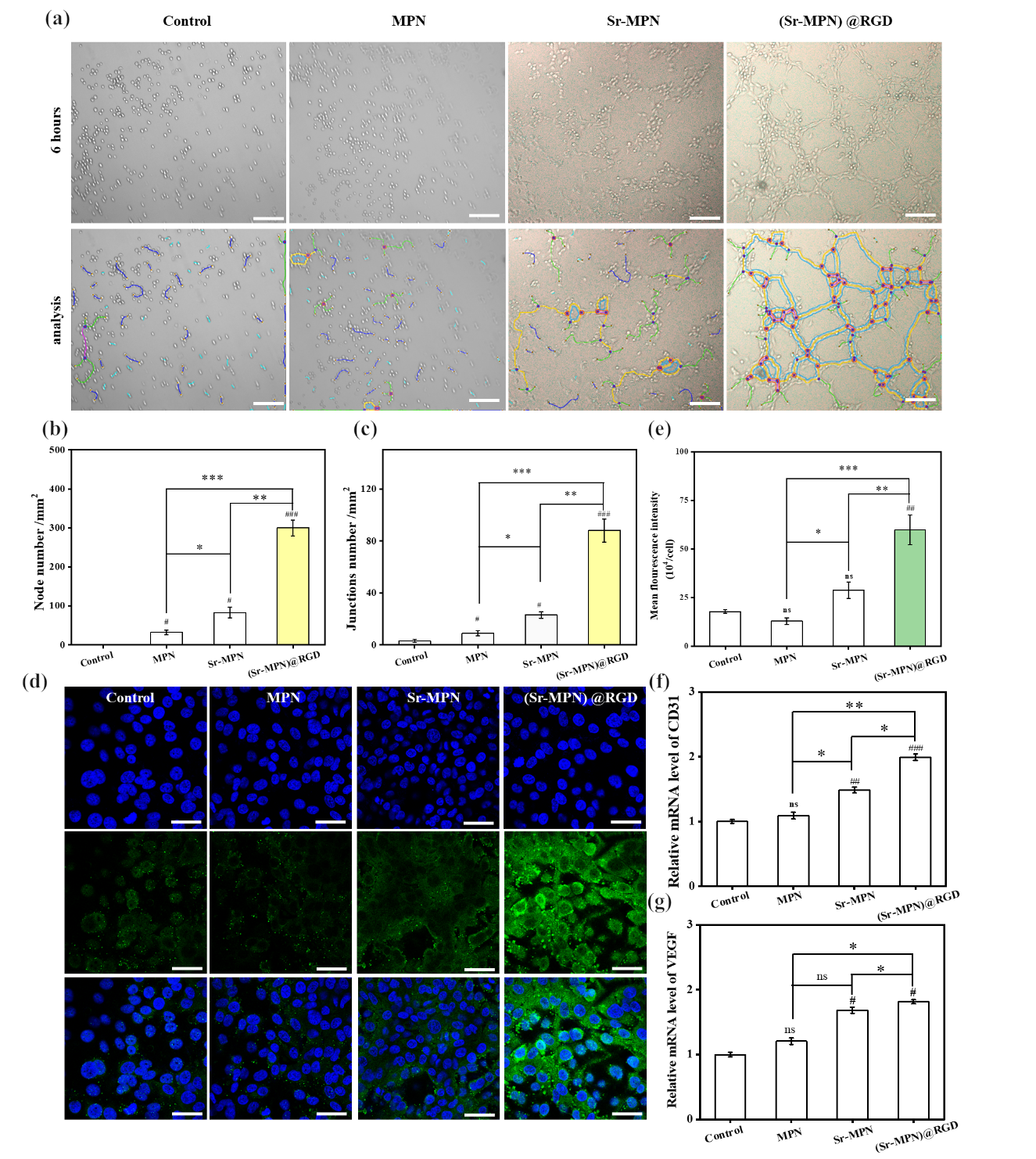
**

**Figure S6. (a)** Vascularization after 6 hours of cellular growth on matrix gel. Statistical analysis in the number of **(b)** nodes and **(c)** junctions from **(a)**. **(d)** The fluorescent images of HUVEC with stained CD31 antibodies. **(e)** The mean fluorescent intensity of CD31 from **(d)**. The expression levels of angiogenesis-related factors **(f)** CD31 and **(g)** VEGF. The scale bar in **(a)** is 300 um and 200 um in **(d)**. N=3, using t-test, no significance noted as "ns," *p < 0.05, **p < 0.01, ***p < 0.001 compared between the two group, and #p < 0.05, ##p < 0.01 or ###p < 0.001 compared with the Control group.


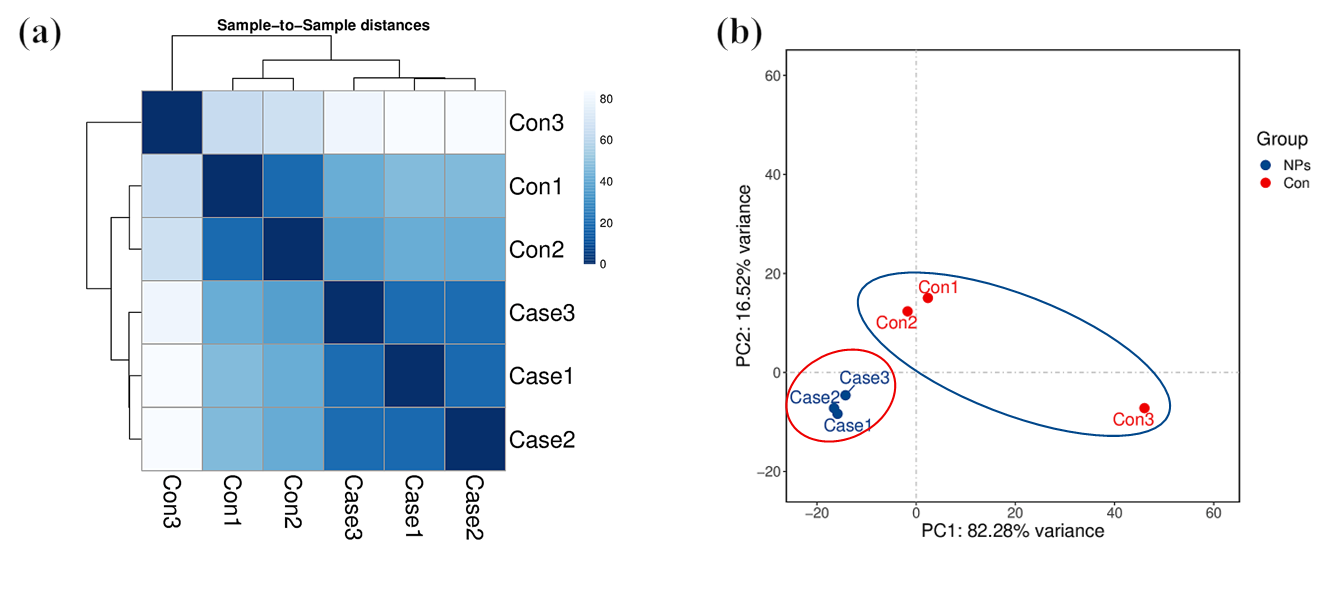


**Figure S7. (a)** Pearson correlation between the three detected samples and **(b)** corresponding principle component analysis.


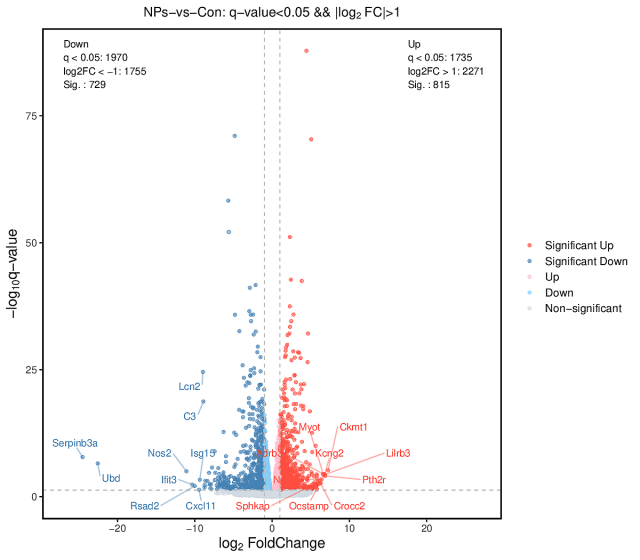


**Figure S8.** Volcano plots of DEGs between the (Sr&Fe-MPN)@RGD group and Ti control group.


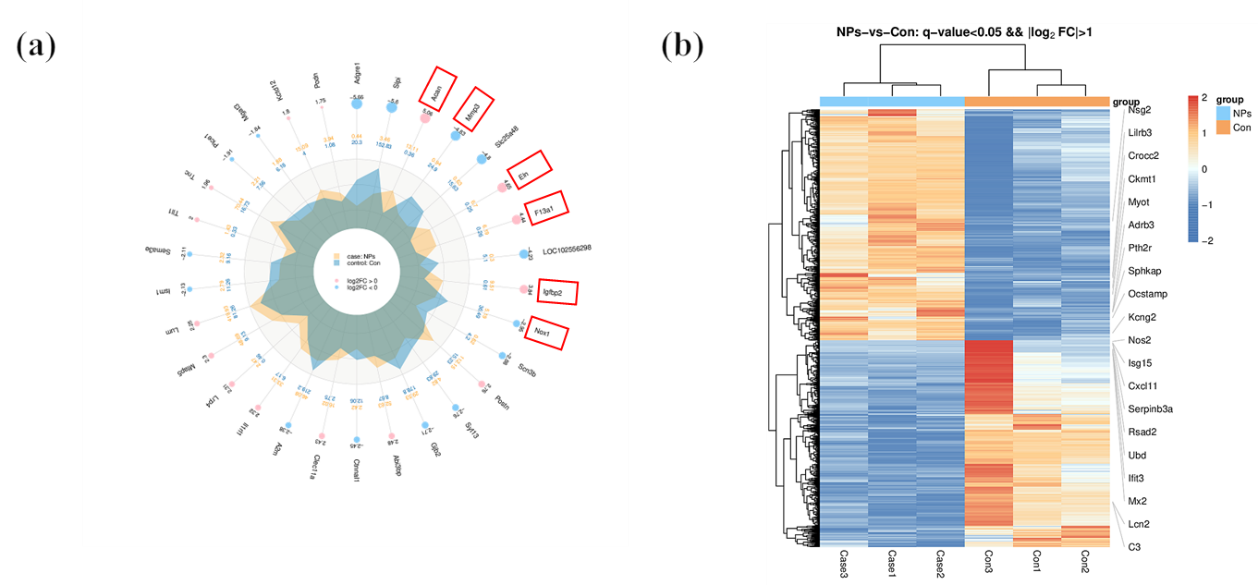


**Figure S9. (a)** Radar chart **(b)** Heatmap of DEGs between the (Sr&Fe-MPN)@RGD group and Ti control group.


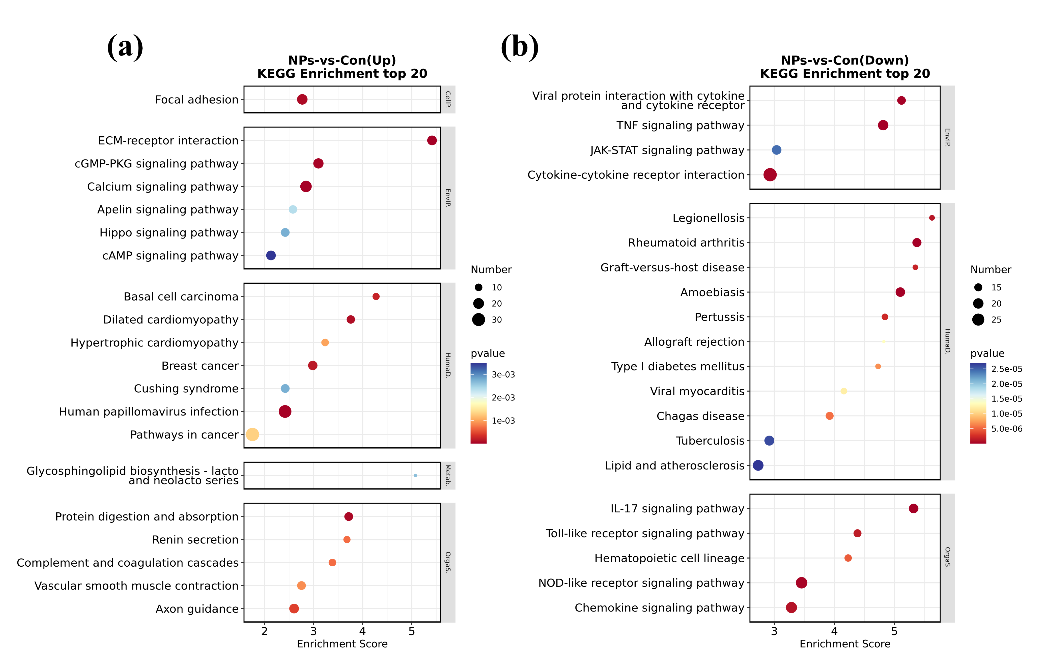


**Figure S10.** KEGG enrichment analysis of **(a)** up-regulated and **(b)** down-regulated genes.

**
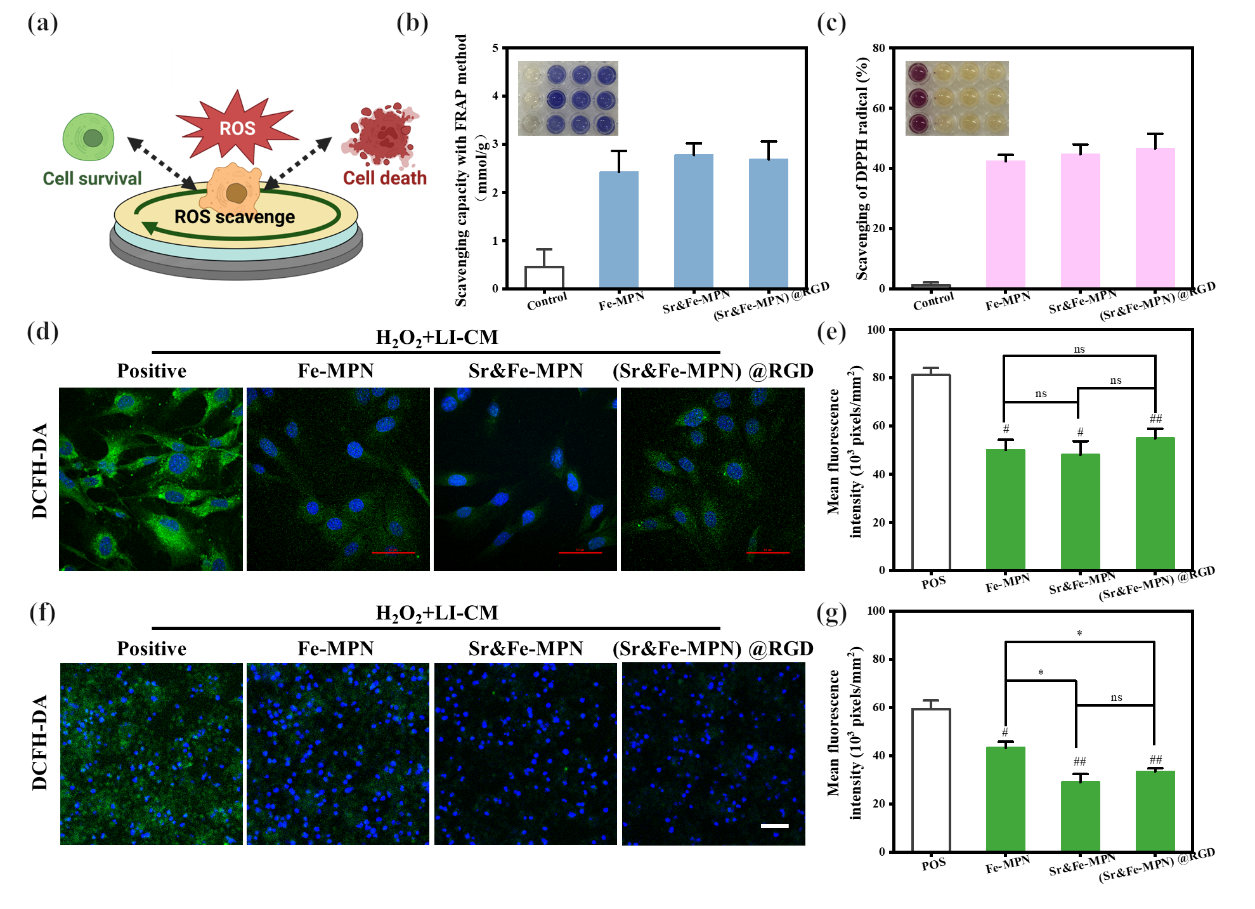
**

**Figure S11.** The anti-radical oxidative stress and anti-apoptotic properties of Fe-MPN, Sr&Fe-MPN and (Sr&Fe-MPN)@RGD coatings in vitro. **(a)** Schematic representation of intracellular ROS and the removal of ROS by the coating to protect the cells. **(b)** The total antioxidant capacity of control, Fe-MPN, Sr&Fe-MPN and (Sr&Fe-MPN)@RGD coatings were determined by FRAP method. **(c)** Free radical scavenging capacity of four coatings were determined by DPPH assay. Fluorescence microscopy images of **(d)** BMSCs and **(f)** BMMs stained with DCFH-DA, mean fluorescence intensity per square mm of **(e)** BMSCs and **(g)** BMMs cultured on different coatings with H_2_O_2_ + LI-CM treatment. Scale bars in **(d)**, **(f)** are 50 µm. N=3, no significance noted as "ns," *p<0.05, **p<0.01, ***p<0.001 compared between the two group, and #p < 0.05, ##p < 0.01 or ###p < 0.001 compared with the Control group or POS group, using t-test.


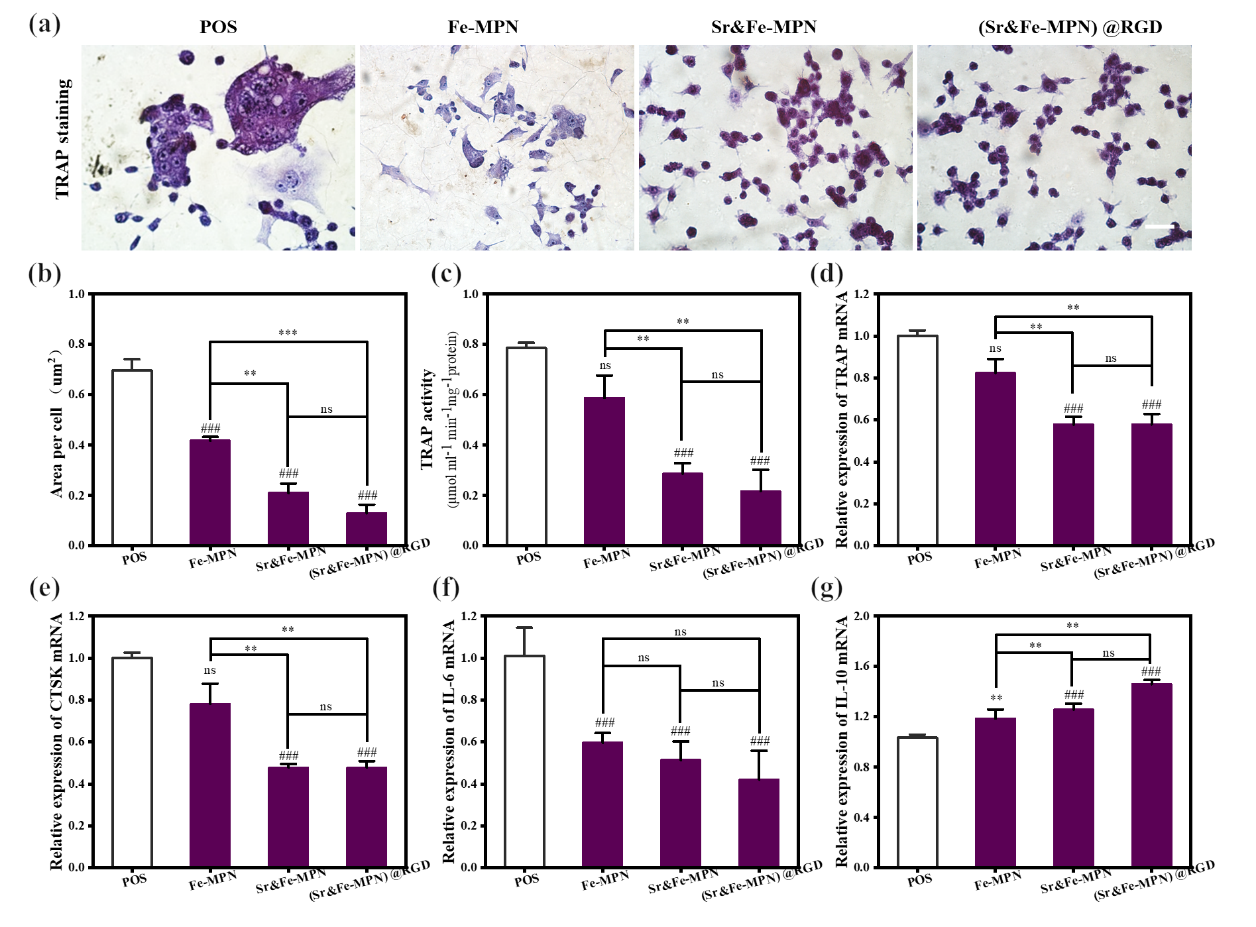


**Figure S12**. Osteoclast differentiation of BMMs on different modified substrates. **(a)** TRAP staining of BMMs after 5 days of co-culture with different coatings in α-MEM medium containing 25 μg/L M-CSF+50 μg/L RANKL. **(b)** Multi-nucleated cell count analysis of **(a)**. **(c)** Quantitative analysis of TRAP activity. Relative mRNA expression of **(d)** TRAP, **(e)** CTSK, **(f)** IL-6 and **(g)** IL-10 genes in BMMs cultured on different modified substrates with different coatings in α-MEM medium containing 25 μg/L M-CSF+50 μg/L RANKL for 5 days. Scale bars in **(a)** are 200 µm. N=3, no significance noted as "ns," *p<0.05, **p<0.01, ***p<0.001 compared between the two group, and #p < 0.05, ##p < 0.01 or ###p < 0.001 compared with the POS control group, using t-test.

**Table S1.** Primer sets (Rattus) used for quantitative real-time PCR ^[1-3]^.

| **RNA template** | **Forward primer (5′-3′)** | **Reverse primer (5′-3′)** |
| --- | --- | --- |
| β-Actin | CTCATGCCATCCTGCGTCTG | GGCAGTGGCCATCTCTTGCT |
| CD86 | TGTTTCCGTGGAGACGCAAG | TTGAGCCTTTGTAAATGGGCA |
| CD206 | CTCTGTTCAGCTATTGGACGC | CGGAATTTCTGGGATTCAGCTTC |
| iNOS | GTTCTCAGCCCAACAATACAAGA | GTGGACGGGTCGATGTCAC |
| IL-1β | GCAACTGTTCCTGAACTCAACT | ATCTTTTGGGGTCCGTCAACT |
| IL-6 | TACCACTCCCAACAGACCTG | ACTCCCAGAAGACCAGAGGAA |
| IL-10 | TTTCAAACAAAGGACCAG | GGATCATTTCCGATAAGG |
| TGF-β | CTCCCGTGGCTTCTAGTGC | GCCTTAGTTTGGACAGGATCTG |
| Col-1 | GGCTGGTTCACTGGGATTG | CCGTTTGCATAGTTTGCTCTGG |
| OCN | GGACCATCTTTCTGCTCACTCTG | TTCACTACCTTATTGCCCTCCTG |
| OPN | TCTGATGAGACCGTCACTGC | AGGTCCTCATCTGTGGCATC |
| ON | TGAGGACGGTGCAGAGGA | TGGTGGCAAAGAAGTGGC |
| CTSK | GCACCCTTAGTCTTCCGCTC | GGTCATATAGCCGCCTCCAC |
| TRAP | CTGGAGTGCACGATGCCAGCGACA | TCCGTGCTCGGCGATGGACCAGA |

**Table S2**. Elemental analysis of different coatings detected by XPS

| **Coatings** | **C%** | **O%** | **Fe%** | **Sr%** | **N%** |
| --- | --- | --- | --- | --- | --- |
| Fe-MPN | 64.14 | 31.28 | 4.58 | - | - |
| Sr&Fe-MPN | 64.97 | 30.46 | 3.68 | 0.89 | - |
| (Sr&Fe-MPN)@RGD | 66.59 | 24.63 | 1.31 | 0.58 | 6.89 |

[1] X. Fu, P. Liu, D. Zhao, B. Yuan, Z. Xiao, Y. Zhou, X. Yang, X. Zhu, C. Tu, X. Zhang, Effects of Nanotopography Regulation and Silicon Doping on Angiogenic and Osteogenic Activities of Hydroxyapatite Coating on Titanium Implant, Int J Nanomedicine, 15 (2020) 4171-4189. <https://doi.org/10.2147/IJN.S252936>.

[2] Y. Dai, L. Chu, Z. Luo, T. Tang, H. Wu, F. Wang, S. Mei, J. Wei, X. Wang, X. Shang, Effects of a Coating of Nano Silicon Nitride on Porous Polyetheretherketone on Behaviors of MC3T3-E1 Cells in Vitro and Vascularization and Osteogenesis in Vivo, ACS Biomater Sci Eng, 5 (2019) 6425-6435. <https://doi.org/10.1021/acsbiomaterials.9b00605>.

[3] M. Wu, F. Chen, H. Liu, P. Wu, Z. Yang, Z. Zhang, J. Su, L. Cai, Y. Zhang, Bioinspired sandwich-like hybrid surface functionalized scaffold capable of regulating osteogenesis, angiogenesis, and osteoclastogenesis for robust bone regeneration, Mater Today Bio, 17 (2022) 100458. <https://doi.org/10.1016/j.mtbio.2022.100458>.
